# Cu/Mn bimetallic catalysis enables carbonylative Suzuki–Miyaura coupling with unactivated alkyl electrophiles†
†Electronic supplementary information (ESI) available. See DOI: 10.1039/c7sc01170a



**DOI:** 10.1039/c7sc01170a

**Published:** 2017-03-31

**Authors:** Dominic R. Pye, Li-Jie Cheng, Neal P. Mankad

**Affiliations:** a Department of Chemistry , University of Illinois at Chicago , 845 W. Taylor St. , Chicago , IL 60607 , USA . Email: npm@uic.edu

## Abstract

A bimetallic system consisting of Cu-carbene and Mn-carbonyl co-catalysts was employed for carbonylative C–C coupling of arylboronic esters with alkyl halides, allowing for the convergent synthesis of ketones. The system operates under mild conditions and exhibits complementary reactivity to Pd catalysis. The method is compatible with a wide range of arylboronic ester nucleophiles and proceeds smoothly for both primary and secondary alkyl iodide electrophiles. Preliminary mechanistic experiments corroborate a hypothetical catalytic mechanism consisting of co-dependent cycles wherein the Cu-carbene co-catalyst engages in transmetallation to generate an organocopper nucleophile, while the Mn-carbonyl co-catalyst activates the alkyl halide electrophile by single-electron transfer and then undergoes reversible carbonylation to generate an acylmanganese electrophile. The two cycles then intersect with a heterobimetallic, product-releasing C–C coupling step.

## Abstract 2

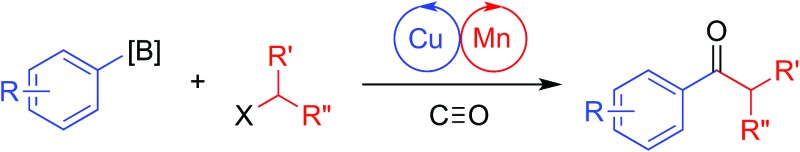
A bimetallic system consisting of Cu-carbene and Mn-carbonyl co-catalysts was employed for carbonylative C–C coupling of arylboronic esters with alkyl halides, allowing for the convergent synthesis of ketones.

## Introduction

**Fig. 1:**
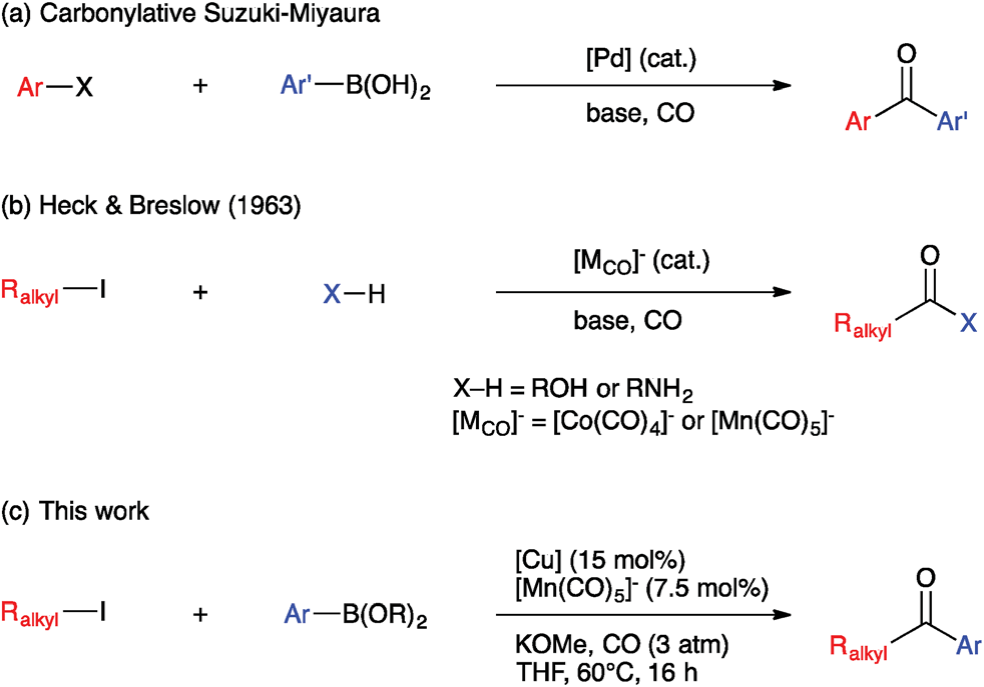
Previous work on (a) Pd-catalyzed Suzuki–Miyaura and (b) base metal-catalyzed heterocarbonylation reactions; (c) this work on base metal-catalyzed carbonylative C–C coupling with alkyl iodides.

Ketones are ubiquitous functional groups in organic molecules and materials, and furthermore their established reaction chemistry can be used to introduce other heteroatom-containing functional groups at late stages of complex syntheses. A convergent method for constructing ketones from simple building blocks is Pd-catalyzed carbonylative C–C coupling, such as the carbonylative Suzuki–Miyaura, Mizoroki–Heck, and Sonogashira coupling reactions.^1–3^ These Pd-catalyzed carbonylations have been long established for C(sp^2^)-hybridized electrophiles, *i.e.* aryl and vinyl halides (Fig. 1a). The use of C(sp^3^)-hybridized electrophiles, *i.e.* alkyl halides, has been reported for specialized cases lacking β-hydrogens,^4–9^ but cases involving alkylpalladium intermediates susceptible to β-hydride elimination have been more challenging to solve.^10–12^ Ryu recently reported a Pd-catalyzed method that solves this problem in a general way but requires irradiation with a Xe lamp to generate alkyl radicals that undergo carbonylation at high pressure (45 atm).^13^ Non-carbonylative Pd-catalyzed reactions to generate alkyl-substituted ketones involve arylcarboxylic acid derivatives as electrophiles^14^ and include the Liebeskind–Srogl^15^ and Fukuyama^16^ coupling reactions. Both carbonylative^17,18^ and non-carbonylative^19^ Ni-catalyzed reductive coupling reactions to generate alkyl-substituted ketones also have been reported.

**Scheme 1:**
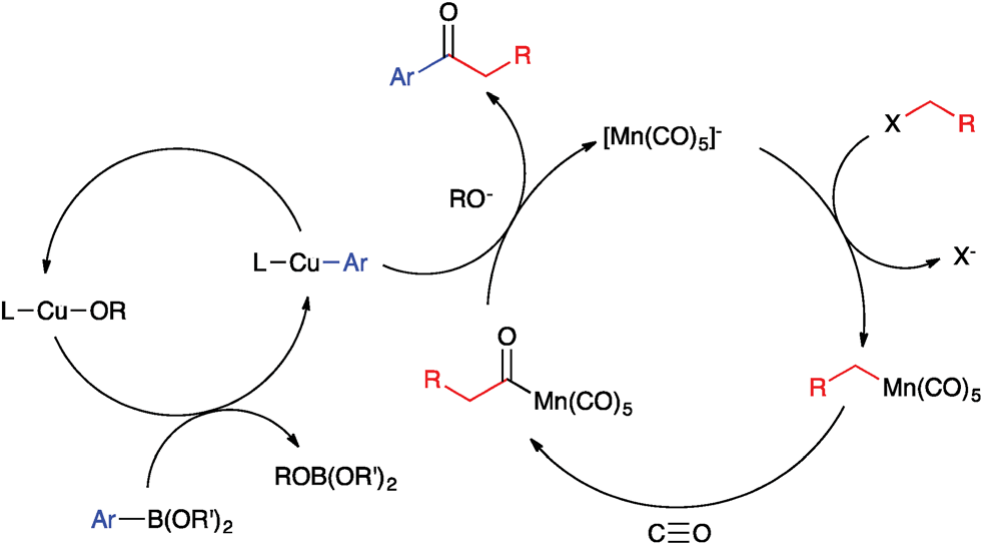
Bimetallic mechanism for carbonylative Suzuki–Miyaura coupling.

Base metal carbonylate complexes are efficient catalysts for heterocarbonylation reactions of alkyl halides. Heck and Breslow first reported the use of Na[Co(CO)_4_] as a catalyst for the formation of esters from alkyl halides, CO, and alcohols over 50 years ago.^20^ Since then, the formation of esters and amides *via* carbonylation of alkyl halides in the presence of an appropriate nucleophile, *i.e.* an alcohol or amine, has been studied using both Co- and Mn-carbonyl pre-catalysts (Fig. 1b).^21,22^ Coates has extended this chemistry to include epoxide carbonylation using a bifunctional catalysis approach.^23,24^ There has not, however, been a suitable carbon nucleophile identified to participate in carbonylative C–C coupling chemistry to form ketones within such systems. Many carbon nucleophiles, such as Grignard reagents, that would be sufficiently nucleophilic to participate in the desired catalytic C–C coupling processes would also react with the ketone products, thus destroying the target molecules. Organocopper nucleophiles are used extensively for 1,4-addition to α,β-unsaturated ketones due to their resistance towards 1,2-addition to carbonyl groups.^25^ Thus, we hypothesized that a heterobimetallic system consisting of organocopper intermediates in combination with Co or Mn carbonylates would enable carbonylative C–C coupling with alkyl halides to generate ketones, without then consuming the resulting ketone products (Fig. 1c). Our hypothetical mechanism for a carbonylative Suzuki–Miyaura reaction, then, consists of a Heck–Breslow cycle for alkyl halide carbonylation combined with a Cu cycle that generates catalytic quantities of an arylcopper species from a mild arylboronic ester nucleophile (Scheme 1). The two co-dependent cycles intersect with a heterobimetallic C–C coupling step between an arylcopper nucleophile and a metal-acyl electrophile. This mechanistic paradigm represents a new frontier in bimetallic catalysis for C–C coupling,^26^ which otherwise typically involves catalytic generation of an organometallic nucleophile that undergoes transmetallation with a catalytically generated Pd(ii) electrophile,^27–34^ thus still requiring use of Pd catalysis and its inherent limitations.

## Results and discussion

As a starting point, we investigated the reaction of 4-tolylboronic acid neopentyl glycol ester (**1a**) and 1-iodooctane (**2a**) under an atmosphere of carbon monoxide. Initial experimentation indicated that the desired ketone (**3**) formed in 40% yield in THF solvent at 60 °C in the presence of KOMe base (1.5 equiv.) and catalytic amounts (10 mol%) of (IPr)CuCl and Na[Mn(CO)_5_] (Table 1, Entry 1). Lower yields were obtained with bulkier alkoxide bases or with tricyclohexylphosphine in place of the IPr carbene. The same result was obtained by using catalytic (IPr)Cu–Mn(CO)_5_, which is expected to assemble upon mixing the Cu and Mn co-catalysts,^35^ and so optimization was continued using separate pre-catalysts rather than with Cu/Mn-bonded heterobimetallic complexes. Substituting IPr for ^Cl^IPr gave a slight increase in yield of **3** to 45%, and performing the reaction under modestly higher CO pressure (3 atm) raised the yield to 59% (Entries 2–3). A screen of numerous copper-carbene co-catalysts at this pressure (see ESI†) identified a number that gave yields in the 70–75% range, and so we continued with commercially available and easily synthesized (IPr)CuCl (Entry 4). Altering the pre-catalyst loadings to 15% (IPr)CuCl and 7.5% Na[Mn(CO)_5_] was found empirically to increase yield of **3** to 88% (Entry 5). No catalysis was observed when omitting either the Cu co-catalyst or the Mn co-catalyst from the reaction (Entries 6–7), thus verifying the need for bimetallic catalysis. With regard to the nucleophilic coupling partner, efficient reactivity also was observed using the pinacol ester (**1b**) rather than **1a**, while poor reactivity was observed with the unprotected boronic acid (**1c**) (Entries 8–9).

**Table 1:** Optimization of Cu/Mn-catalyzed carbonylative C–C coupling

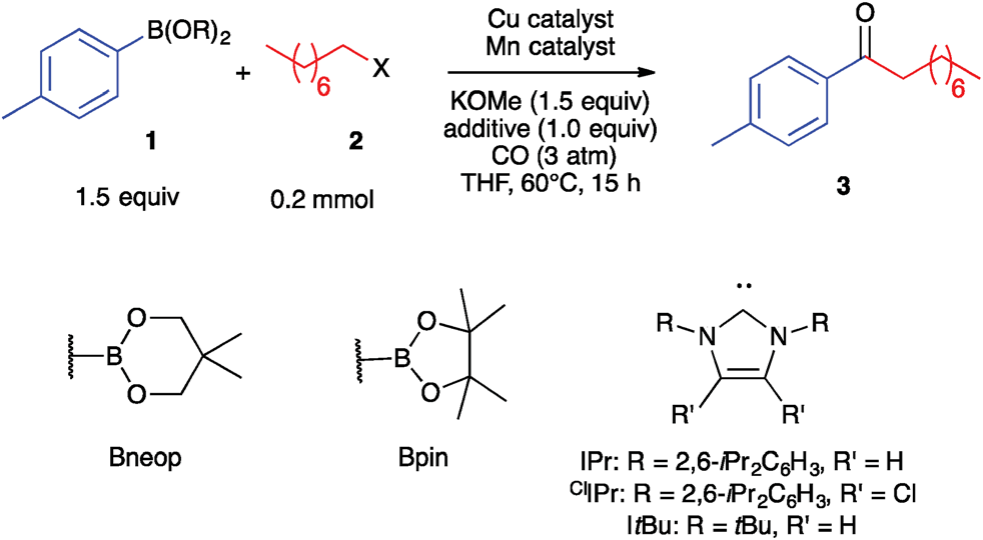					
Entry	B(OR)_2_	X	Cu catalyst	Mn catalyst	Yield of **3** (%)
1^*a*^	Bneop (**1a**)	I (**2a**)	10% (IPr)CuCl	10% Na[Mn(CO)_5_]	40^*b*^
2^*a*^	Bneop (**1a**)	I (**2a**)	10% (^Cl^IPr)CuCl	10% Na[Mn(CO)_5_]	45^*b*^
3	Bneop (**1a**)	I (**2a**)	10% (^Cl^IPr)CuCl	10% Na[Mn(CO)_5_]	59^*b*^
4	Bneop (**1a**)	I (**2a**)	10% (IPr)CuCl	10% Na[Mn(CO)_5_]	73^*b*^
5	Bneop (**1a**)	I (**2a**)	15% (IPr)CuCl	7.5% Na[Mn(CO)_5_]	88^*b*^ (70)^*c*^
6	Bneop (**1a**)	I (**2a**)	15% (IPr)CuCl	None	5^*b*^
7	Bneop (**1a**)	I (**2a**)	None	7.5% Na[Mn(CO)_5_]	0^*b*^
8	Bpin (**1b**)	I (**2a**)	15% (IPr)CuCl	7.5% Na[Mn(CO)_5_]	69^*c*^
9	B(OH)_2_ (**1c**)	I (**2a**)	15% (IPr)CuCl	7.5% Na[Mn(CO)_5_]	42^*c*^
10	Bneop (**1a**)	Br (**2b**)	15% (IPr)CuCl	7.5% Na[Mn(CO)_5_]	13^*c*^
11^*d*^	Bneop (**1a**)	Br (**2b**)	15% (IPr)CuCl	7.5% Na[Mn(CO)_5_]	66^*c*^
12	Bneop (**1a**)	OTs (**2c**)	15% (IPr)CuCl	7.5% Na[Mn(CO)_5_]	0^*c*^
13^*d*^	Bneop (**1a**)	OTs (**2c**)	15% (IPr)CuCl	7.5% Na[Mn(CO)_5_]	74^*c*^
14	Bneop (**1a**)	I (**2a**)	15% (IPr)CuCl	3.8% Mn_2_(CO)_10_	56^*c*^

^*a*^ *p*_CO_ = 1 atm.

^*b*^Yield determined by NMR analysis (1,3,5-trimethoxybenzene internal standard).

^*c*^Yield determined by GC analysis (decane internal standard).

^*d*^Additive = Bu_4_N^+^I^–^.

Ketone **3** did not form in high yield when 1-bromooctane (**2b**) or 1-octyltosylate (**2c**) were used in place of **2a**, but reactivity was restored when these reactions were run in the presence of stoichiometric tetrabutylammonium iodide, presumably *via* the *in situ* formation of **2a** (Entries 10–13). Lastly, ketone **3** was formed with only slightly compromised yield when using 0.5 Mn_2_(CO)_10_ in place of Na[Mn(CO)_5_] (Entry 14). This result is noteworthy for the use of (IPr)CuCl and Mn_2_(CO)_10_ co-catalysts, both of which are commercially available and stable to air and moisture. We verified that no non-carbonylative Suzuki–Miyaura (alkylated arene), Heck–Breslow (ester), or Williamson (ether) products were formed in these reactions. Rather, the mass balance consists of unidentified decomposition products.

In order to investigate functional group compatibility relevant to the synthesis of complex and functionally dense ketone products, we used the carbonylative formation of **3** to conduct a Glorius robustness screen.^36^ The formation of **3** was found to be robust towards a remarkable range of remote functional groups (Fig. S1†). Strongly electrophilic groups such as aldehydes, esters, and nitriles are tolerated, demonstrating the judicious choice of organocopper nucleophiles in this system. Nucleophilic groups such as a sulfur heterocycle and an unprotected primary amine also were tolerated. Intriguingly, an aryl bromide additive did not have measureable impact on the formation of **3** and was inert under the reaction conditions. This observation is particularly noteworthy, as typical Pd catalysts would readily activate C(sp^2^)–Br bonds. Only mild poisoning was observed with protic additives such as alcohols and terminal alkynes. Pyridine and *N*-methyl benzamide were the only additives definitively not tolerated in this robustness screen because they inhibited the reaction or were unstable under the conditions, respectively. Collectively, these results demonstrate that the Cu/Mn-catalyzed carbonylative coupling reaction is ideal both for late-stage functionalization and for the synthesis of ketones bearing latent synthetic handles useful for further elaboration, with only a small number of exceptions. It is noteworthy that many of the functional groups tolerated here would be incompatible with traditional Friedel–Crafts acylation conditions.

**Fig. 2:**
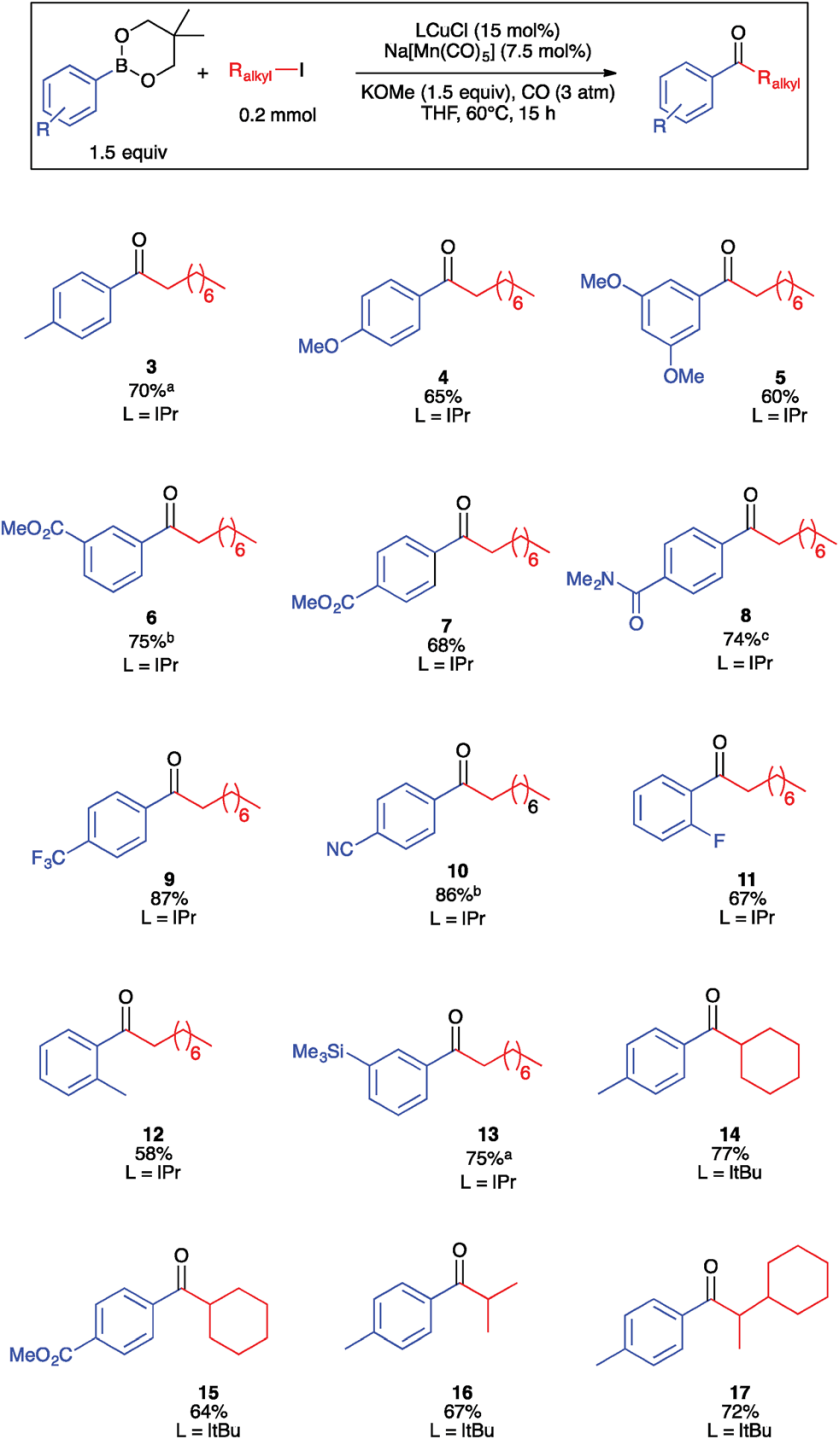
Substrate scope of Cu/Mn-catalyzed carbonylative Suzuki–Miyaura coupling. Yields determined by product isolation unless otherwise indicated. ^a^GC yield (decane internal standard). ^b^From arylboronic acid pinacol ester. ^c^Isolated as 2 : 1 mixture with *N*,*N*-dimethylbenzamide.

Guided by the results of the robustness screen, we next conducted substrate scope studies to examine steric and electron constraints on the coupling mechanism (Fig. 2). Electron-rich, electron-poor, and sterically encumbered arylboronic esters underwent carbonylative coupling with **2a** efficiently to generate ketones **3–13** in moderate to high yields. It is noteworthy that several of these ketones represent aromatic regioisomers that would be unavailable by Friedel–Crafts acylation. A secondary alkyl iodide, iodocyclohexane, underwent carbonylative coupling with **1a** in only 10% yield under the optimized conditions. We reasoned that formation of cyclohexyl-substituted ketone **14** may be slow due to the relevant secondary manganese-acyl intermediate being more sterically hindered, and therefore less electrophilic, than the corresponding primary intermediate derived from **2a**. Using the smaller and more electron-donating I*t*Bu in place of IPr provided **14** in excellent yield. Under these conditions, ketone **15**, which is a synthetic precursor to a glucagon receptor modulator marketed by Pfizer,^37^ also was synthesized in high yield. Acyclic secondary alkyl iodides also reacted smoothly, allowing for the isolation of ketones **16** and **17**. The formation of chiral **17** opens the opportunity for future development of asymmetric catalysis for the enantioselective formation of ketones with α-stereocenters, a possibility that would be challenging for Pd-catalyzed carbonylation. Ketones derived from benzyl or allyl electrophiles were not observed, presumably due to their instability under the basic reaction conditions. Another limitation of the method is that ketones derived from vinyl- or alkynylboronic esters were not observed, presumably because they are susceptible to conjugate addition under the reaction conditions. Lastly, various heteroarylboronic ester nucleophiles that we examined did not undergo productive carbonylative coupling with **2a**.

**Fig. 3:**
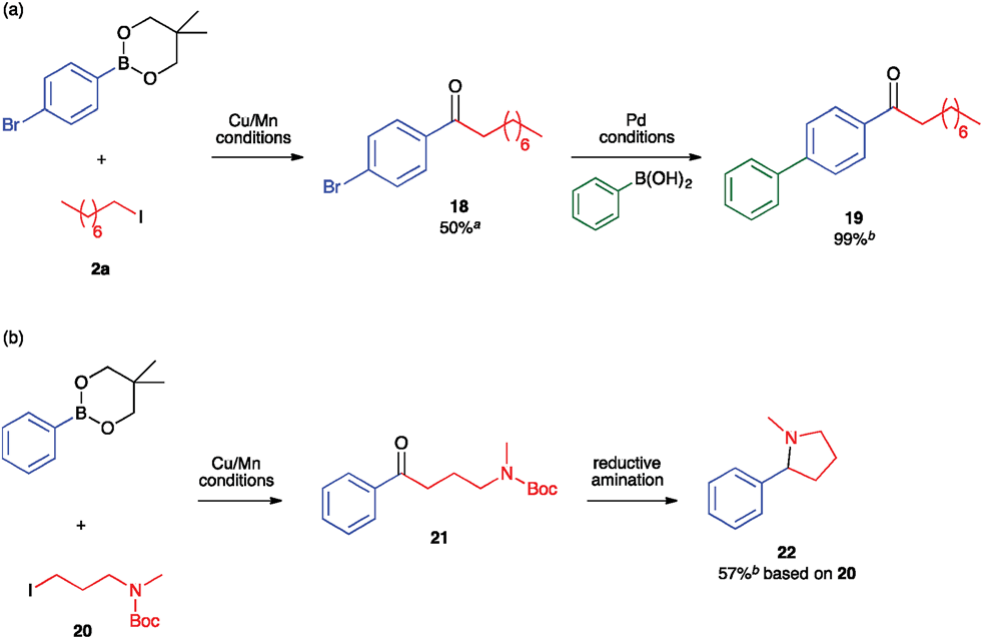
Reaction sequences demonstrating (a) orthogonality of Cu/Mn catalysis and Pd catalysis; (b) relevance of Cu/Mn catalysis to heterocycle synthesis. Cu/Mn conditions: catalytic conditions: alkyl iodide (0.2 mmol), arylboronic ester (1.5 eq.), KOMe (1.5 eq.), (IPr)CuCl (15 mol%), Na[Mn(CO)_5_] (7.5 mol%), THF (5 mL), CO (3 atm), 60 °C, 15 h. Pd conditions: aryl bromide (0.5 mmol), arylboronic acid (2.0 eq.), K_2_CO_3_ (2.0 eq.), PdCl_2_(PPh_3_)_2_ (10 mol%), toluene : H_2_O (10 : 1, 5.5 mL), 100 °C, 21 h. Reductive amination conditions: (i) CF_3_CO_2_H (0.5 mL), CH_2_Cl_2_ (3 mL), 0 °C, 2 h, (ii) NaBH_4_ (4 eq.), MeOH (5 mL), 1 h. ^a^Isolated yield. ^b^Yield determined by NMR analysis (1,3,5-trimethoxybenzene internal standard).

To leverage the assets of our method, we sought both to synthesize ketones bearing synthetic handles for further elaboration and to construct ketones that can serve as synthons for privileged structures relevant to drug molecules. First, to demonstrate the orthogonality of Cu/Mn catalysis and Pd catalysis, we conducted the Cu/Mn-catalyzed carbonylative coupling reaction with an arylboronic ester nucleophile containing an aryl-bromide linkage that would be unstable under Pd-catalyzed conditions (Fig. 3a). Isolation of the resulting ketone **18** proceeded smoothly without activation of the C(sp^2^)–Br position. Further elaboration at that position was then demonstrated through its use as the electrophilic component of a traditional Pd-catalyzed Suzuki–Miyaura coupling reaction, which provided the 4-biphenyl ketone **19** quantitatively. Second, to demonstrate utility towards the synthesis of drug molecules, we conducted the Cu/Mn-catalyzed carbonylative coupling with 3-aminoalkyl iodide **20**, which provided the 3-aminoalkyl ketone **21** (Fig. 3b). Subjecting a crude sample of **21** to standard *N*-Boc deprotection and reductive amination conditions provided the 2-arylpyrrolidine product **22** in 57% yield from **20**. The C(sp^3^)-C(sp^2^) linkage α to the nitrogen in **22** was installed by Cu/Mn-catalyzed C–C coupling. Pyrrolidines and related C(sp^3^)-rich heterocycles are known to be privileged core structures in drug molecule candidates, and the installation of aromatic substituents in the position α to the heteroatom in such structures by coupling methods is a longstanding challenge actively being pursued by several research groups.^38–42^ Here, the construction of such a motif is enabled by the unique reactivity of the Cu/Mn-catalyzed coupling reaction towards C(sp^3^)-hybridized substrates.

**Fig. 4:**
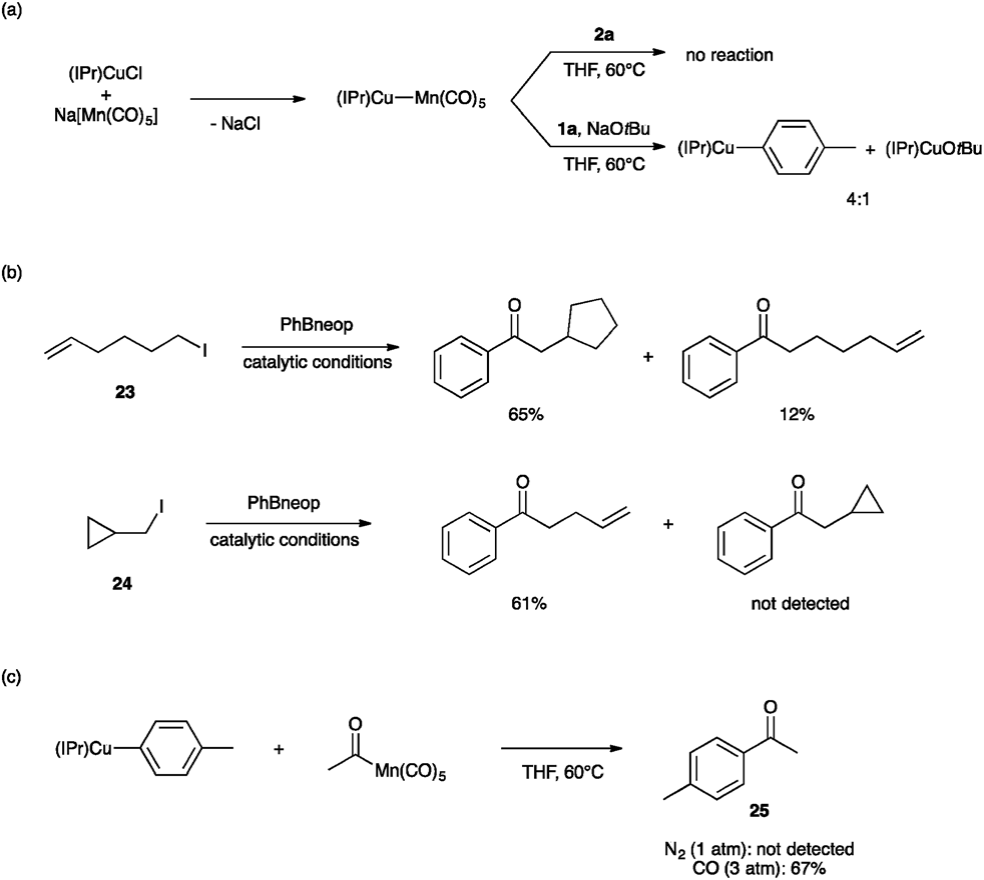
Reactivity studies to probe the catalytic mechanism. Yields determined by NMR analysis (1,3,5-trimethoxybenzene internal standard). Catalytic conditions: alkyl iodide (0.2 mmol), arylboronic ester (1.5 eq.), KOMe (1.5 eq.), (IPr)CuCl (15 mol%), Na[Mn(CO)_5_] (7.5 mol%), THF (5 mL), CO (3 atm), 60 °C, 15 h.

Lastly, we sought to probe the viability of our hypothetical mechanism through stoichiometric reactivity studies. First, because (IPr)Cu–Mn(CO)_5_ is expected to assemble upon mixing (IPr)CuCl and Na[Mn(CO)_5_],^35^ we sought to establish its role in the bimetallic catalysis (Fig. 4a). The metal–metal bonded complex (IPr)Cu–Mn(CO)_5_ was found to be unreactive towards **2a** in THF at 60 °C. On the other hand, exposing this metal–metal bonded complex to NaO*t*Bu and **1a** under the same conditions led to a mixture of (IPr)CuO*t*Bu, (IPr)Cu(tol) (tol = *p*-tolyl), and presumably Na[Mn(CO)_5_]. Based on these observations, we propose that the metal–metal bonded complex (IPr)Cu–Mn(CO)_5_ is not an on-cycle catalytic intermediate capable of activating any of the coupling partners directly, but rather it is unstable under the basic conditions of catalysis towards formation of (IPr)CuOMe and the “unmasked” K[Mn(CO)_5_]. Alkylation of [Mn(CO)_5_]^–^ by iodoalkanes is well known^20^ and is thought to proceed by a single-electron transfer mechanism.^21,22^ Consistent with this proposal, we observed radical cyclization/ring-opening behavior with the radical clock iodoalkanes **23** and **24** (Fig. 4b). Lastly, the ketone-generating, bimetallic C–C coupling step between the arylcopper and acylmanganese intermediates has no precedent in the literature. In order to establish feasibility of this mechanistically novel C–C coupling step, we examined reactivity between isolated samples of (IPr)Cu(tol) and MeC(O)Mn(CO)_5_ (Fig. 4c). When the experiment was conducted in THF at 60 °C under N_2_ atmosphere, no 4-methylacetophenone (**25**) was observed. However, when the same reaction was conducted under an atmosphere of CO (3 atm), the expected ketone product **25** was formed in 67% yield. These results are consistent with the product-releasing C–C bond formation being viable but having to compete with de-insertion of CO, thus requiring the application of CO pressure. This stoichiometric C–C coupling reaction demonstrates the key role of a novel heterobimetallic C–C bond-forming step in the catalytic generation of ketones.

## Conclusions

Bimetallic catalysis with earth-abundant Cu and Mn was leveraged to discover C–C coupling chemistry that complements existing methods with single-site Pd catalysis. Conceptually, the bimetallic scheme is novel in the context of transformations featuring two co-dependent catalytic metals in that it does not utilize Pd. A limitation of Pd catalysis, namely the inability to efficiently carbonylate C(sp^3^)-hybridized electrophiles, was thus overcome.
