# Supplementary material for: Cu/Mn bimetallic catalysis enables carbonylative Suzuki–Miyaura coupling with unactivated alkyl electrophiles
Source: Chem Sci. 2017 Mar 31;8(7):4750–5. doi: 10.1039/c7sc01170a (PMC5585854; doi:10.1039/c7sc01170a)

Electronic Supplementary Material (ESI) for Chemical Science.
This journal is © The Royal Society of Chemistry 2017

Supporting Information for

Cu/Mn Bimetallic Catalysis Enables Carbonylative Suzuki-Miyaura Coupling with Unactivated Alkyl Electrophiles

Dominic R. Pye, Li-Jie Cheng, Neal P. Mankad*

Department of Chemistry, University of Illinois at Chicago, 845 W. Taylor St., Chicago, IL 60607, USA

* email: npm@uic.edu

Contents:

General Considerations	S2
General Procedure & Optimization	S2
Robustness Screen	S5
Ketone Synthesis & Characterization Data	S7
Stoichiometric Reactions	S15
References	S16
Spectral Data	S17

General Considerations

Unless otherwise specified, all reactions and manipulations were performed under purified N_2 in a glovebox or using standard Schlenk line techniques. Glassware was oven-dried prior to use. THF was sparged with argon and dried using a Glass Contour Solvent System built by Pure Process Technology, LLC. CO gas was purchased from Praxair at a purity of 99.99% (4.0RS research grade) and used directly from the cylinder. Unless otherwise specified, all chemicals were purchased from commercial sources and used without further purification. Literature methods were used to synthesize $Na[Mn(CO)_5]$,[1] $[MeC(O)Mn(CO)_5]$,[2] IPrCuCl, SIMesCuCl, ItBuCuCl, IMesCuCl,[3] ^{Cl}IPrCuCl, ^{Cl}IMesCuCl,[4] CaacCuCl,[5] arylboronic acid neopentylglycol esters,[6] and 1-octyltosylate[7]. Flash column chromatography was performed using SiliaFlash F60 40-63µm (230-400 mesh) 60Å Irregular Silica Gels.

Reactions at greater than atmospheric pressure were conducted in either a Parr 4621 General Purpose Pressure Reactor or an Andrews Glass 3 oz footed glass reaction vessel. A fitted, aluminium insert was custom-made for the Parr reactor that could hold up to nine 22-mL scintillation vials for running reactions in parallel.

NMR spectra were recorded at ambient temperature using Bruker Avance DPX-400 and Bruker Avance DRX-500 spectrometers. ^{1}H NMR and $^{13}C\{^{1}H\}$ NMR chemical shifts were referenced to residual solvent. GC analysis was performed on a Shimadzu GC-2010 Plus. HRMS analyses were performed on JEOL GC-Mate **II** mass spectrometer with electron impact (EI).

General Procedure A: Pressurisation of reaction mixtures with carbon monoxide.

Taking carbon monoxide directly from the cylinder, the reactor was pressurised to three atmospheres whilst stirring at 300 rpm. After five minutes the pressure was released, resealed and let stir for 1 minute, after which any residual pressure was released. This process was repeated once, and then the reactor was pressurised to three atmospheres carbon monoxide, let stir under pressure for 10 minutes, after which the reaction vessel was sealed and heated to the required temperature. Note that, due to the high thermal mass of the Parr pressure reactor, around one hour was required for the internal temperature to reach 60 °C.

Optimisation of the formation of 4-tolylnonanone from 4-tolylboronic acid neopentylglycol ester, 1-iodooctane and carbon monoxide (Table 1).

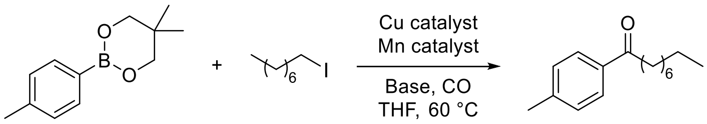

To a 22-mL scintillation vial, or directly into the 3 oz glass reactor, was added the appropriate (NHC)CuCl, $Na[Mn(CO)_5]$, 4-tolylboronic acid neopentyl glycol ester, KOMe, 1-iodooctane (36 µL, 0.2 mmol), 1,3,5-trimethoxybenznene (16.8 mg, 0.1 mmol, internal standard) and THF (5 mL). The vial was capped with a septum containing lid, and the septa pierced with a needle. The vial was placed within a solid, fitted, aluminium rack within a Parr pressure reactor and pressurised according to

General Procedure A. The reaction was then heated at 60 °C for 16 hours. Upon completion of the reaction an aliquot of the reaction mixture was taken and filtered through a silica plug, eluting with diethyl ether, and the sample was analysed by NMR for product.

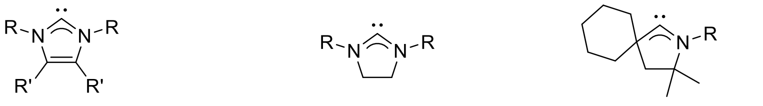

IPr: R = 2,6-*i*$Pr_2C_6H_3$, R' = H
IMes: R = 2,4,6-$Me_3C_6H_3$, R' = H
[Cl]IPr: R = 2,6-*i*$Pr_2C_6H_3$, R' = Cl
[Cl]Mes: R = 2,4,6-$Me_3C_6H_3$, R' = Cl
I*t*Bu: R = *t*Bu, R' = H

SIMes: R = 2,4,6-$Me_3C_6H_3$

CAAC: R = 2,6-*i*$Pr_2C_6H_3$

Catalyst (mol%)	Base (eq)	Nucleophile (eq)	Pressure (atm)	Yield (%)
IPrCu[Mn(CO)$_5$] (10)	NaOtBu (1.5)	tolBneop (1.5)	1	10
	KOMe (1.5)		1	40
[Cl]IPrCuCl (10) + Na[Mn(CO)$_5$] (10)			1	45
			6	55
			3	59
IPrCuCl (10) + Na[Mn(CO)$_5$] (10)			3	73
	(2)	(2)	3	61
	(1.25)	(2)	3	60
	(2)	(1.25)	3	66
	(1.25)	(1.25)	3	60
IPrCuCl (7.5) + Na[Mn(CO)$_5$] (7.5)	(1.5)	(1.5)	3	67
IPrCuCl (15) + Na[Mn(CO)$_5$] (10)			3	73
IPrCuCl (10) + Na[Mn(CO)$_5$] (15)			3	55
IPrCuCl (20) + Na[Mn(CO)$_5$] (10)			3	74
IPrCuCl (20) + Na[Mn(CO)$_5$] (7.5)			3	75
IPrCuCl (20) + Na[Mn(CO)$_5$] (5)			3	74
IPrCuCl (15) + Na[Mn(CO)$_5$] (10)			3	77
IPrCuCl (15) + Na[Mn(CO)$_5$] (7.5)			3	89
IPrCuCl (15) + Na[Mn(CO)$_5$] (5)			3	84
(CAAC)CuCl (15) + Na[Mn(CO)$_5$] (7.5)			3	78
[Cl]IMesCuCl (15) + Na[Mn(CO)$_5$]			3	77

(7.5)				
SIMesCuCl (15) + Na[Mn(CO)$_5$] (7.5)			3	76
I*t*BuCuCl (15) + Na[Mn(CO)$_5$] (7.5)			3	87
IMesCuCl (15) + Na[Mn(CO)$_5$] (7.5)			3	83

Optimisation of the formation of 4-tolylcyclohexyl ketone from 4-tolylboronic acid neopentylglycol ester, iodocyclohexane and carbon monoxide (Table 1).

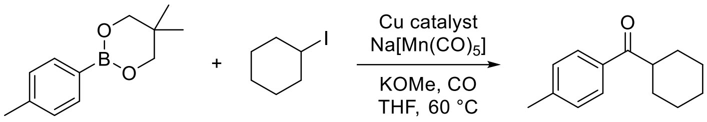

9

To a 22-mL scintillation vial, or directly into the 3 oz glass reactor, was added the appropriate (NHC)CuCl (0.03 mmol), Na[Mn(CO)$_5$] (3.3 mg, 0.015 mmol), 4-tolylboronic acid neopentyl glycol ester (61 mg, 0.3 mmol), KOMe (21 mg, 0.3 mmol) iodocyclohexane (26 µL, 0.2 mmol), 1,3,5-trimethoxybenznene (16.8 mg, 0.1 mmol, internal standard) and THF (5 mL). The vial was capped with a septum containing lid, and the septa pierced with a needle. The vial was placed within a solid, fitted, aluminium rack within the Parr pressure reactor and pressurised according to General Procedure A. The reaction was then heated at 60 °C for 16 hours. Upon completion of the reaction an aliquot of the reaction mixture was taken and filtered through a silica plug, eluting with diethyl ether, and the sample was analysed by NMR for product.

Catalyst (mol%)	Yield (%)
IPrCuCl (15) + Na[Mn(CO)$_5$] (7.5)	10
(CAAC)CuCl (15) + Na[Mn(CO)$_5$] (7.5)	40
^{Cl}IMesCuCl (15) + Na[Mn(CO)$_5$] (7.5)	45
SIMesCuCl (15) + Na[Mn(CO)$_5$] (7.5)	55
ItBuCuCl (15) + Na[Mn(CO)$_5$] (7.5)	95
IMesCuCl (15) + Na[Mn(CO)$_5$] (7.5)	73

Robustness screen (Figure 2).

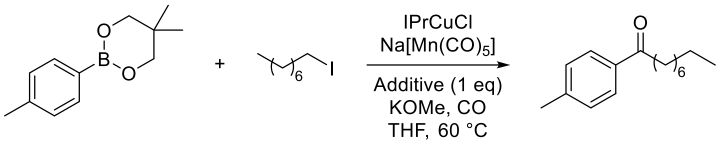

According to the method set out by Collins and Glorius.[8]

To a 22-mL scintillation vial was added IPrCuCl (14.6 mg, 0.03 mmol), Na[Mn(CO)$_5$] (3.3 mg, 0.015 mmol), 4-tolylboronic acid neopentyl glycol ester (61 mg, 0.3 mmol), 1-iodooctane (36 µL, 0.2 mmol), a functional additive (0.2 mmol) and THF (5 mL). The vial was capped with a septum containing lid, and the septa pierced with a needle. The vial was placed within a solid, fitted, aluminium rack within a Parr pressure reactor and pressurised according to General Procedure A. The reaction was then heated at 60°C for 16 hours. Upon completion of the reaction decane (39 µL, 0.2 mmol) was added and an aliquot was taken and filtered through a silica plug, eluting with diethyl ether, and the sample was analysed by GC for product and functional additive.

Functional additive	Functional additive retained (%)	Yield (%)
none	-	70
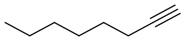	92	52
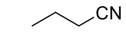	99	70
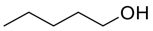	69	56
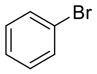	91	71
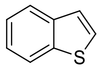	78	72
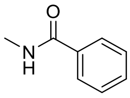	9	65
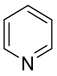	99	9
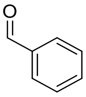	91	66
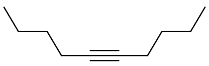	73	70
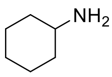	99	68
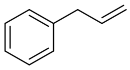	93	69
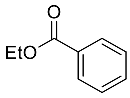	82	68

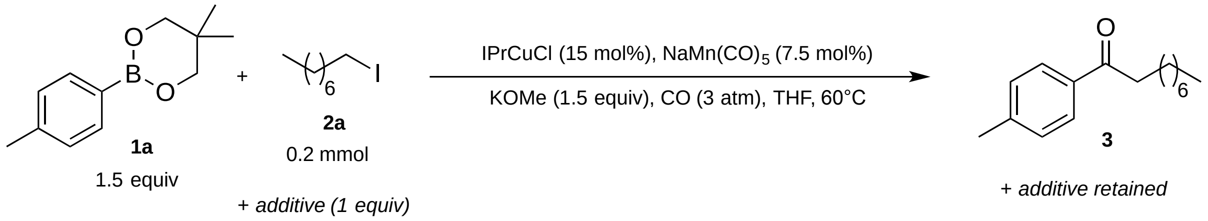

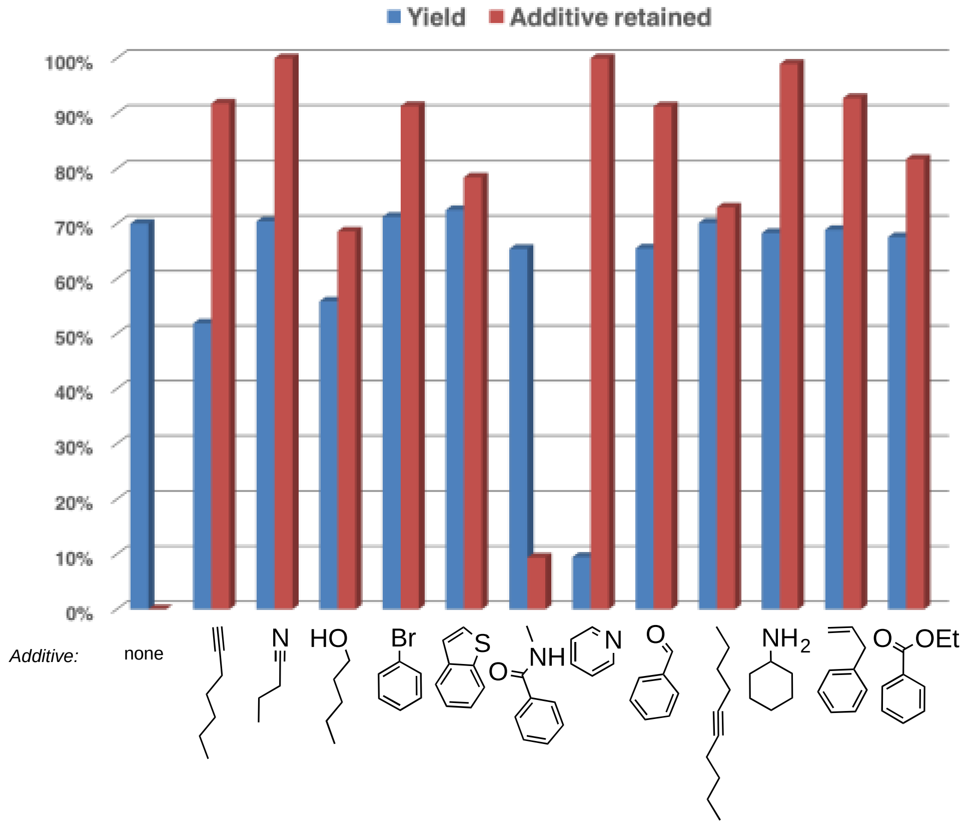

Figure S1. Robustness screening data showing wide functional group tolerance.

The carbonylative formation of ketones from alkyl halides (Figures 2 and 3).

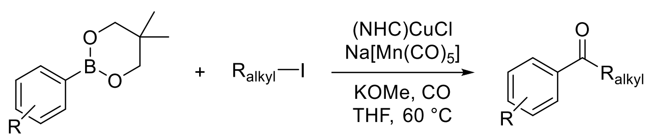

For GC or NMR yields:

To a 22-mL scintillation vial, or directly into the 3 oz glass reactor, was added KOMe (21 mg, 0.3 mmol), the appropriate (NHC)CuCl (0.03 mmol), Na[Mn(CO)$_5$] (3.3 mg, 0.015 mmol), arylboronic acid neopentyl glycol ester (0.3 mmol), alkyl iodide (0.2 mmol) and THF (5 mL). The vial was capped with a septum containing lid, and the septa pierced with a needle. The vial was placed within a solid, fitted, aluminium rack within a Parr pressure reactor and pressurised according to General Procedure A. The reaction was then heated at 60 °C for 16 hours. Upon completion of the reaction an internal standard was added (1,3,5-trimethoxybenzene – NMR analysis, decane – GC analysis). A sample of the reaction mixture was taken and filtered through a silica plug, eluting with diethyl ether, and the sample was analysed for product.

Isolated yields:

(4-Methoxyphenyl)-1-nonanone, 4 (Figure 2).

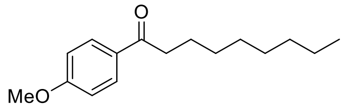

Into the 3 oz glass reactor was added KOMe (210 mg, 3 mmol), IPrCuCl (146 mg, 0.3 mmol), Na[Mn(CO)$_5$] (33 mg, 0.15 mmol), 4-methoxyphenylboronic acid neopentyl glycol ester (660 mg, 3 mmol), 1-iodooctane (361 µL, 2 mmol) and THF (50 mL). The reaction vessel was pressurised according to General Procedure A and heated to 60 °C in an oil bath for 16 hours. Upon completion of the reaction the solvent was removed under reduced pressure, and the crude reaction mixture taken up in hexane. This suspension was filtered through a fine grade glass frit, and concentrated under reduced pressure. The resulting yellow oil was purified by flash column chromatography (2% diethyl ether in hexanes) to give **4** as a white solid (323 mg, 65% yield). NMR data was found to match previously reported values.[10]

^{1}H NMR (500 MHz, CDCl$_3$) δ = 7.95 (d, *J* = 8.5 Hz, 2 H), 6.94 (d, *J* = 8.5 Hz, 2 H), 3.87 (s, 3 H), 2.91 (t, *J* = 7.5 Hz, 2 H), 1.72 (quin, *J* = 7.5 Hz, 2 H), 1.42 - 1.22 (m, 10 H), 0.88 (t, *J* = 6.5 Hz, 3 H)

^{13}C NMR (125 MHz, CDCl$_3$) δ = 199.2, 163.3, 130.3, 130.2, 113.6, 55.4, 38.3, 31.8, 29.4, 29.4, 29.2, 24.6, 22.6, 14.1

(3,5-Dimethoxyphenyl)-1-nonanone, 5 (Figure 2)

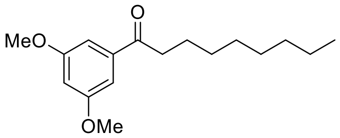

To a 22-mL scintillation vial was added KOMe (52.5 mg, 0.75 mmol), IPrCuCl (36.5 mg, 0.075 mmol), Na[Mn(CO)$_5$] (8.2 mg, 0.0325 mmol), 3,5-dimethoxyphenylboronic acid neopentyl glycol ester (188 mg, 0.75 mmol), KOMe (53 mg, 0.75 mmol) 1-iodooctane (90 µL, 0.5 mmol), and THF (10 mL). The vial was capped with a septum containing lid, and the septa pierced with a needle. The vial was placed within a solid, fitted, aluminium rack within the Parr pressure reactor and pressurised according to General Procedure A. The reaction was then heated at 60 °C for 16 hours. Upon completion of the reaction the solvent was removed under reduced pressure and the resulting mixture was purified by flash column chromatography (5% diethyl ether in hexanes) to give **5** as a white solid (84 mg, 60%).

^{1}H NMR (500 MHz, CDCl$_3$) δ = 7.09 (d, J = 2.0 Hz, 2 H), 6.64 (s, 1 H), 3.83 (s, 6 H), 2.91 (t, J = 7.5 Hz, 2 H), 1.75 - 1.67 (m, 2 H), 1.41 - 1.24 (m, 10 H), 0.88 (t, J = 6.5 Hz, 3 H).

^{13}C NMR (125 MHz, CDCl$_3$) δ = 200.2, 160.8, 139.0, 105.9, 105.0, 55.5, 38.7, 31.8, 29.4, 29.3, 29.1, 24.4, 22.6, 14.0.

Methyl-(3-nonanoyl)benzoate, 6 (Figure 2)

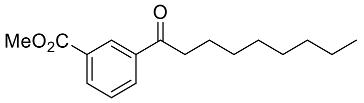

To a 22-mL scintillation vial was added IPrCuCl (14.6 mg, 0.075 mmol), Na[Mn(CO)$_5$] (8.3 mg, 0.038 mmol), 3-methoxycarbonylbenzene boronic acid pinacol ester (197 mg, 0.75 mmol), KOMe (53 mg, 0.75 mmol) 1-iodooctane (90 µL, 0.5 mmol), and THF (10 mL). The vial was capped with a septum containing lid, and the septa pierced with a needle. The vial was placed within a solid, fitted, aluminium rack within the Parr pressure reactor and pressurised according to General Procedure A. The reaction was then heated at 60 °C for 16 hours. Upon completion of the reaction the solvent was removed under reduced pressure and the resulting mixture was purified by flash column chromatography (8% diethyl ether in hexanes) to give **6** as a pale-yellow oil (104 mg, 75%).

^{1}H NMR (500 MHz, CDCl$_3$) δ = 8.57 (s, 1 H), 8.20 (d, *J* = 7.9 Hz, 1 H), 8.14 (d, *J* = 7.3 Hz, 1 H), 7.53 (t, *J* = 7.6 Hz, 1 H), 3.94 (s, 3 H), 2.98 (t, *J* = 7.3 Hz, 2 H), 1.73 (quin, *J* = 7.9 Hz, 2 H), 1.42 - 1.21 (m, 10 H), 0.86 (t, *J* = 6.7 Hz, 3 H).

^{13}C NMR (125 MHz, CDCl$_3$) δ = 199.5, 166.2, 137.2, 133.5, 132.1, 130.5, 129.1, 128.7, 52.3, 38.6, 31.8, 29.4, 29.2, 29.1, 24.1, 22.6, 14.0.

HRMS (EI) Calcd. for $C_{17}H_{24}O_3$ ([M]+): 276.1720; Found: 276.1734.

Methyl-(4-nonanoyl)benzoate, 7 (Figure 2).

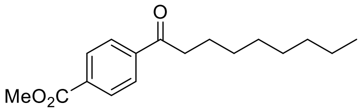

Into the 3 oz glass reactor was added KOMe (210 mg, 3 mmol), IPrCuCl (146 mg, 0.3 mmol), Na[Mn(CO)$_5$] (33 mg, 0.15 mmol), 4-methoxycarbonylbenzeneboronic acid neopentyl glycol ester (660 mg, 3 mmol), 1-iodooctane (744 µL, 2 mmol) and THF (50 mL). The reaction vessel was pressurised according to General Procedure A and heated to 60 °C in an oil bath for 16 hours. Upon completion of the reaction the solvent was removed under reduced pressure, and the crude reaction mixture taken up in hexane. This suspension was filtered through a fine grade glass frit, and concentrated under reduced pressure. The resulting yellow oil was purified by flash column chromatography (3% diethyl ether in hexanes) to give **7** as a white solid (376 mg, 68% yield).

^{1}H NMR (500 MHz, CDCl$_3$) δ = 8.12 (d, *J* = 8.0 Hz, 2 H), 8.00 (d, *J* = 8.0 Hz, 2 H), 3.95 (s, 3 H), 2.99 (t, *J* = 7.5 Hz, 2 H), 1.74 (quin, *J* = 7.5 Hz, 2 H), 1.45 - 1.20 (m, 10 H), 0.88 (t, *J* = 6.0 Hz, 3 H).

^{13}C NMR (125 MHz, CDCl$_3$) δ = 200.0, 166.2, 140.3, 133.6, 129.8, 127.9, 52.4, 39.0, 31.8, 29.4, 29.3, 29.1, 24.1, 22.6, 14.1.

N,N-Dimethyl-4-nonanoylbenzamide, 8 (Figure 2)

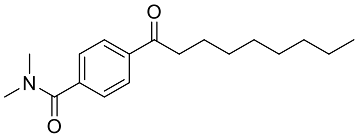

To a 22 mL scintillation vial was added KOMe (52.5 mg, 0.75 mmol), IPrCuCl (36.5 mg, 0.075 mmol), Na[Mn(CO)$_5$] (8.2 mg, 0.0325 mmol), 4-(N,N-dimethylaminocarbonyl)benzeneboronic acid neopentyl glycol ester (145 mg, 0.75 mmol), KOMe (53 mg, 0.75 mmol) 1-iodooctane (90 µL, 0.5 mmol), and THF (10 mL). The vial was capped with a septum containing lid, and the septa pierced with a needle. The vial was placed within a solid, fitted, aluminium rack within the Parr pressure reactor and pressurised according to General Procedure A. The reaction was then heated at 60 °C for 16 hours. Upon completion of the reaction the solvent was removed under reduced pressure and the resulting mixture was purified by flash column chromatography (50% ethyl acetate in hexanes) to give a white solid which was found to contain **8** and N,N-dimethylbenzamide (the product of protodeborylation of the boronic acid) in a ratio of approximately 2:1 according to ^{1}H NMR (135 mg total, 74% yield of **8**).

^{1}H NMR (500 MHz, CDCl$_3$) δ = 7.95 (d, *J* = 8.5 Hz, 2 H), 7.46 (d, *J* = 8.5 Hz, 2 H), 2.98 - 2.89 (m, 6 H), 1.70 (quin, *J* = 7.5 Hz, 2 H), 1.40 - 1.18 (m, 10 H), 0.84 (t, *J* = 7.0 Hz, 3 H).

^{13}C NMR (125 MHz, CDCl$_3$) δ = 199.8, 170.5, 140.4, 137.5, 128.0, 126.9, 39.2, 38.7, 35.1, 31.7, 29.3, 29.2, 29.0, 24.2, 22.5, 14.0.

HRMS (EI) Calcd. for $C_{18}H_{27}O_2N$ ([M]$^+$): 289.2037; Found: 289.2037.

(4-Trifluoromethylphenyl)-1-nonanone, 9 (Figure 2)

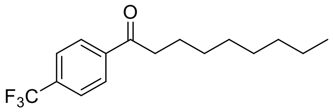

To a 22-mL scintillation vial was added KOMe (52.5 mg, 0.75 mmol), IPrCuCl (36.5 mg, 0.075 mmol), Na[Mn(CO)$_5$] (8.2 mg, 0.0325 mmol), 4-trifluoromethylphenylboronic acid neopentyl glycol ester (188 mg, 0.75 mmol), KOMe (53 mg, 0.75 mmol) 1-iodooctane (90 µL, 0.5 mmol), and THF (10 mL). The vial was capped with a septum containing lid, and the septa pierced with a needle. The vial was placed within a solid, fitted, aluminium rack within the Parr pressure reactor and pressurised according to General Procedure A. The reaction was then heated at 60 °C for 16 hours. Upon completion of the reaction the solvent was removed under reduced pressure and the resulting mixture was purified by flash column chromatography (2% diethyl ether in hexanes) to give **9** as a white solid (124 mg, 87%).

^{1}H NMR (500 MHz, CDCl$_3$) δ = 8.11 - 8.03 (m, J = 8.0 Hz, 2 H), 7.76 - 7.69 (m, J = 8.0 Hz, 2 H), 2.99 (t, J = 7.5 Hz, 2 H), 1.75 (quin, J = 7.5 Hz, 2 H), 1.44 - 1.23 (m, 10 H), 0.89 (t, J = 6.5 Hz, 3 H).

^{13}C NMR (125 MHz, CDCl$_3$) δ = 199.5, 139.7, 134.2 (q, *J* = 33 Hz), 128.3, 125.6 (q, *J* = 126 Hz), 123.6 (q, *J* = 273 Hz), 38.9, 31.8, 29.4, 29.3, 29.1, 24.1, 22.6, 14.1.

4-Nonanoylbenzonitrile, **10 (Figure 2)**

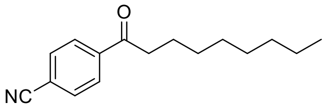

To a 22-mL scintillation vial was added KOMe (52.5 mg, 0.75 mmol), IPrCuCl (36.5 mg, 0.075 mmol), Na[Mn(CO)$_5$] (8.2 mg, 0.0325 mmol), 4-cyanophenylboronic acid pinacol ester (172 mg, 0.75 mmol), KOMe (53 mg, 0.75 mmol) 1-iodooctane (90 µL, 0.5 mmol), and THF (10 mL). The vial was capped with a septum containing lid, and the septa pierced with a needle. The vial was placed within a solid, fitted, aluminium rack within the Parr pressure reactor and pressurised according to General Procedure A. The reaction was then heated at 60 °C for 16 hours. Upon completion of the reaction the solvent was removed under reduced pressure and the resulting mixture was purified by flash column chromatography (9% ethyl acetate in hexanes) to give **10** as a white solid (104 mg, 86%).

^{1}H NMR (500 MHz, CDCl$_3$) δ = 8.03 (d, *J* = 8.0 Hz, 2 H), 7.76 (d, *J* = 8.0 Hz, 2 H), 2.97 (t, *J* = 7.5 Hz, 2 H), 1.73 (quin, *J* = 7.5 Hz, 2 H), 1.44 - 1.21 (m, 10 H), 0.87 (t, *J* = 6.5 Hz, 3 H)

^{13}C NMR (125 MHz, CDCl$_3$) δ = 199.0, 139.9, 132.4, 128.4, 117.9, 116.1, 38.8, 31.7, 29.3, 29.1, 29.0, 23.9, 22.6, 14.0

HRMS (EI) Calcd. for $C_{16}H_{21}ON$ ([M]$^+$): 243.1618; Found: 243.1622.

(2-Fluorophenyl)-1-nonanone, 11 (Figure 2)

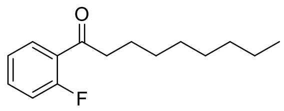

To a 22-mL scintillation vial was added KOMe (52.5 mg, 0.75 mmol), IPrCuCl (36.5 mg, 0.075 mmol), Na[Mn(CO)$_5$] (8.2 mg, 0.0325 mmol), 2-fluorophenylboronic acid neopentyl glycol ester (156 mg, 0.75 mmol), KOMe (53 mg, 0.75 mmol) 1-iodooctane (90 µL, 0.5 mmol), and THF (10 mL). The vial was capped with a septum containing lid, and the septa pierced with a needle. The vial was placed within a solid, fitted, aluminium rack within the Parr pressure reactor and pressurised according to General Procedure A. The reaction was then heated at 60 °C for 16 hours. Upon completion of the reaction the solvent was removed under reduced pressure and the resulting mixture was purified by flash column chromatography (2% diethyl ether in hexanes) to give **11** as a white solid (80 mg, 67%).

^{1}H NMR (500 MHz, CDCl$_3$) δ = 7.84 (dt, *J* = 1.5, 7.5 Hz, 1 H), 7.53 - 7.47 (m, 1 H), 7.22 (t, *J* = 7.5 Hz, 1 H), 7.12 (dd, *J* = 8.0, 10.5 Hz, 1 H), 2.97 (dt, *J* = 3.0, 7.5 Hz, 2 H), 1.71 (quin, *J* = 7.5 Hz, 2 H), 1.41 - 1.21 (m, 10 H), 0.88 (t, *J* = 6.5 Hz, 3 H)

^{13}C NMR (125 MHz, CDCl$_3$) δ = 199.0 (d, *J* = 4 Hz), 161.8 (d, *J* = 254 Hz), 134.2 (d, *J* = 9 Hz), 130.6 (d, *J* = 3 Hz), 125.9 (d, *J* = 13 Hz), 124.3 (d, *J* = 3 Hz), 116.6 (d, *J* = 24 Hz), 43.6 (d, *J* = 7 Hz), 31.8, 29.4, 29.3, 29.1, 24.0 (d, *J* = 2 Hz), 22.6, 14.1

HRMS (EI) Calcd. for $C_{15}H_{21}OF$ ($[M]^+$): 236.1571; Found: 236.1569.

(3-Trimethylsilyl)-1-nonanone, 13 (Figure 2)

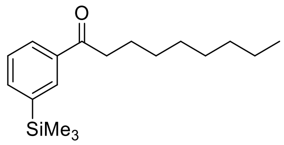

To a 22-mL scintillation vial was added KOMe (52.5 mg, 0.75 mmol), IPrCuCl (36.5 mg, 0.075 mmol), Na[Mn(CO)$_5$] (8.2 mg, 0.0325 mmol), 3-trimethylsilylphenylboronic acid neopentyl glycol ester (197 mg, 0.75 mmol), KOMe (53 mg, 0.75 mmol) 1-iodooctane (90 µL, 0.5 mmol), and THF (10 mL). The vial was capped with a septum containing lid, and the septa pierced with a needle. The vial was placed within a solid, fitted, aluminium rack within the Parr pressure reactor and pressurised according to General Procedure A. The reaction was then heated at 60 °C for 16 hours. Upon completion of the reaction the solvent was removed under reduced pressure and the resulting mixture was purified by flash column chromatography (4% ethyl acetate in hexanes) to give **13** as a white solid (109 mg, 75%).

^{1}H NMR (500 MHz, CDCl$_3$) δ = 8.12 (s, 1 H), 7.93 (d, *J* = 7.5 Hz, 1 H), 7.71 (d, *J* = 7.5 Hz, 1 H), 7.45 (t, *J* = 7.5 Hz, 1 H), 2.98 (t, *J* = 7.5 Hz, 2 H), 1.75 (quin, *J* = 7.5 Hz, 2 H), 1.44 - 1.22 (m, 10 H), 0.89 (t, *J* = 7.0 Hz, 3 H), 0.31 (s, 9 H).

^{13}C NMR (125 MHz, CDCl$_3$) δ = 201.0, 141.2, 137.7, 136.3, 132.7, 128.5, 127.9, 38.6, 31.8, 29.4, 29.4, 29.2, 24.4, 22.6, 14.1, -1.2.

HRMS (EI) Calcd. for $C_{18}H_{30}OSi$ ($[M]^+$): 290.2061; Found: 290.1987.

2-Methyl-1-(4-methylphenyl)-1-propanone, 16 (Figure 2).

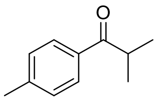

10

To a 22-mL scintillation vial was added ItBuCuCl (8.4 mg, 0.075 mmol), $Na[Mn(CO)_5]$ (8.3 mg, 0.038 mmol), 4-tolylboronic acid neopentyl glycol ester (153 mg, 0.75 mmol), KOMe (53 mg, 0.75 mmol) 2-iodopropane (50 µL, 0.5 mmol), and THF (10 mL). The vial was capped with a septum containing lid, and the septa pierced with a needle. The vial was placed within a solid, fitted, aluminium rack within the Parr pressure reactor and pressurised according to General Procedure A. The reaction was then heated at 60 °C for 16 hours. Upon completion of the reaction the solvent was removed under reduced pressure and the resulting mixture was purified by flash column chromatography (4% diethyl ether in hexanes) to give **16** as a clear oil (54 mg, 67%). NMR data was found to match previously reported values.[11]

^{1}H NMR (500 MHz, $CDCl_3$) δ = 7.87 (d, *J* = 8.0 Hz, 2 H), 7.27 (d, *J* = 8.0 Hz, 2 H), 3.55 (spt, *J* = 6.0 Hz, 1 H), 2.42 (s, 3 H), 1.22 (d, *J* = 7.0 Hz, 6 H).

^{13}C NMR (125 MHz, $CDCl_3$) δ = 204.1, 143.5, 133.7, 129.3, 128.4, 35.2, 21.6, 19.2.

1-Cyclohexyliodoethane.

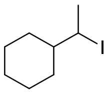

Iodine (4.4 g. 17.4 mmol) was added to a solution of triphenylphosphine (4.6 g, 17.4 mmol) and imidazole (1.18 g, 17.4 mmol) in toluene (80 mL) at room temperature and stirred for 15 minutes. The reaction mixture was then heated to 80°C and 1-cyclohexylethanol (2 mL, 14.5 mL) was added. After 2 hours the reaction mixture cooled to room temperature and washed with saturated aqueous sodium thiosulfate (80 mL). The organic layer was separated, dried ($MgSO_4$) and the solvent removed under reduced pressure. The resultant white solid was extracted with hexane (3 x 50 mL), the extracts combined, concentrated under reduced pressure, and purified by flash column chromatography (hexanes) to give 1-cyclohexyliodoethane as a clear liquid (1.62 g, 47%).

^{1}H NMR (500 MHz, $CDCl_3$) δ = 4.23 (dq, J = 4.0, 7.0 Hz, 1 H), 1.91 (d, J = 7.3 Hz, 3 H), 1.85 (d, J = 10.0 Hz, 1 H), 1.81 - 1.74 (m, 3 H), 1.69 - 1.62 (m, 1 H), 1.36 - 1.22 (m, 2 H), 1.21 - 0.99 (m, 4 H).

^{13}C NMR (125 MHz, $CDCl_3$) δ = 46.5, 40.2, 32.0, 31.4, 26.3, 26.0, 25.8.

HRMS (EI) Calcd. for $C_8H_{15}I$ ($[M]^+$): 238.0213; Found: 238.0218.

2-Cyclohexyl-1-(4-methylphenyl)-1-propanone, 17 (Figure 2).

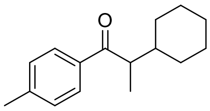

11

To a 22-mL scintillation vial was added ItBuCuCl (8.4 mg, 0.075 mmol), Na[Mn(CO)$_5$] (8.3 mg, 0.038 mmol), 4-tolylboronic acid neopentyl glycol ester (153 mg, 0.75 mmol), KOMe (53 mg, 0.75 mmol) 1-cyclohexyliodoethane (119 mg, 0.5 mmol), and THF (10 mL). The vial was capped with a septum containing lid, and the septa pierced with a needle. The vial was placed within a solid, fitted, aluminium rack within the Parr pressure reactor and pressurised according to General Procedure A. The reaction was then heated at 60 °C for 16 hours. Upon completion of the reaction the solvent was removed under reduced pressure and the resulting mixture was purified by flash column chromatography (3% diethyl ether in hexanes) to give **17** as a clear oil (83 mg, 72%).

^{1}H NMR (500 MHz, CDCl$_3$) δ = 7.86 (d, *J* = 8.0 Hz, 2 H), 7.26 (d, *J* = 8.0 Hz, 2 H), 3.30 (quin, *J* = 7.0 Hz, 1 H), 2.42 (s, 3 H), 1.79 - 1.59 (m, 5 H), 1.29 - 0.92 (m, 9 H).

^{13}C NMR (125 MHz, CDCl$_3$) δ = 204.6, 143.5, 134.9, 129.2, 128.3, 45.8, 40.8, 32.1, 29.3, 26.4, 26.4, 26.3, 21.6, 14.0.

HRMS (EI) Calcd. for $C_{16}H_{22}O$ ([M]$^+$): 230.1665; Found: 230.1679.

(4-Bromophenyl)-1-nonanone, 18, and subsequent Pd coupling (Figure 3a).

To a 22-mL scintillation vial was added KOMe (105 mg, 1.5 mmol), IPrCuCl (73 mg, 0.15 mmol), Na[Mn(CO)$_5$] (15.5 mg, 0.075 mmol), 4-bromobenzeneboronic acid neopentyl glycol ester (403 mg, 1.5 mmol), 1-iodooctane (181 µL, 0.2 mmol) and THF (10 mL). The vial was capped with a septum containing lid, and the septa pierced with a needle. The vial was placed within a solid, fitted, aluminium rack within a Parr pressure reactor and pressurised according to General Procedure A. The reaction was then heated at 60 °C for 16 hours. Purification by column chromatography (1% diethyl ether in hexanes) gave **18** as a white solid (149 mg, 50%). NMR data was found to match previously reported values.[12]

^{1}H NMR (500 MHz, CDCl$_3$) δ = 7.82 (d, *J*=8.5 Hz, 2 H), 7.60 (d, *J*=8.5 Hz, 2 H), 2.92 (t, *J*=7.5 Hz, 2 H), 1.72 (quin, *J*=7.0 Hz, 2 H), 1.42 - 1.21 (m, 10 H), 0.88 (t, *J*=7.5 Hz, 3 H).

^{13}C NMR (125 MHz, CDCl$_3$) δ = 199.5, 135.7, 131.8, 129.6, 127.9, 38.6, 31.8, 29.4, 29.3, 29.1, 24.3, 22.6, 14.1.

To a 22-mL scintillation vial was added **18** (149 mg, 0.5 mmol), phenylboronic acid (122 mg, 1 mmol), potassium carbonate (138 mg, 1 mmol), $[PdCl_2(PPh_3)_2]$ (35 mg, 0.05 mmol), 1,3,5-trimethoxybenzene (84.1 mg, 0.5 mmol, internal standard), toluene (5 mL) and water (0.5 mL). The vial was sealed with a foil lined cap and heated at 100 °C for 21 hours. The reaction mixture was then cooled to room temperature, and aliquot was taken and filtered through a plug of silica, eluting with diethyl ether. The solvent was then removed under reduced pressure, and the residue taken up in $CDCl_3$ and analysed by ^{1}H NMR. **19** was found to be present in 99% yield. See Appendix 1 for ^{1}H NMR spectrum.

Synthesis of 1-methyl-2-phenylpiperidine, 22 (Figure 3b).

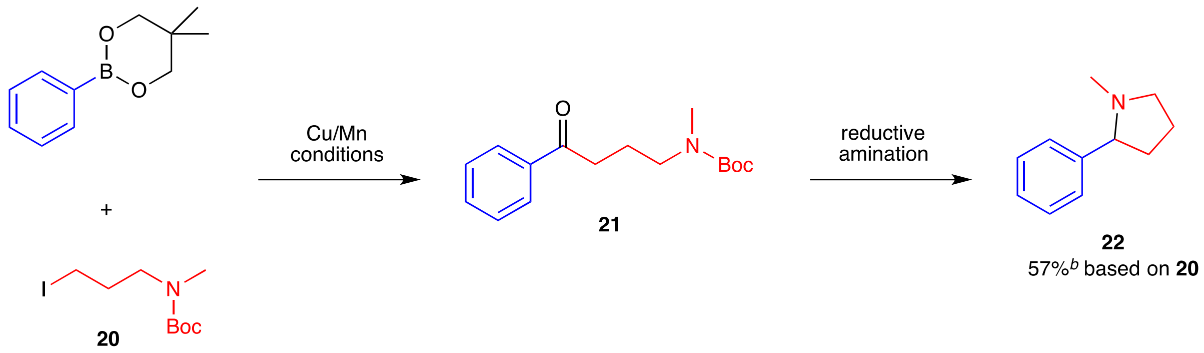

To a 22-mL scintillation vial was added KOMe (52.5 mg, 0.75 mmol), IPrCuCl (36.5 mg, 0.075 mmol), $Na[Mn(CO)_5]$ (8.2 mg, 0.0325 mmol), phenylboronic acid neopentyl glycol ester (143 mg, 0.75 mmol), **20** (150 mg, 0.5 mmol) and THF (10 mL). The vial was capped with a septum containing lid, and the septa pierced with a needle. The vial was placed within a solid, fitted, aluminium rack within a Parr pressure reactor and pressurised according to General Procedure A. The reaction was then heated at 60 °C for 16 hours. The solvent was then removed, and purified by column chromatography (30% diethyl ether in hexanes) to give **21** as a clear oil (113 mg, 70% purity, 70% yield). The mixture containing **21** was taken up in DCM (3 mL) cooled to 0 °C, and trifluoroacetic acid (0.5 mL) was added dropwise. The reaction was maintained at 0°C with stirring for two hours, before the volatiles were removed under reduced pressure. The resulting gum was taken up in methanol (5 mL), cooled to 0°C, and sodium borohydride (76 mg, 2 mmol) was added. The reaction was allowed to warm to room temperature and stirred for one hour. 1,3,5-trimethoxybenzene (42.0 mg, 0.25 mmol, internal standard) was added and the solvent was removed under reduced pressure. The crude mixture was taken up in $CDCl_3$ and analysed by ^{1}H NMR for product. **22** was found to be present in 57% yield with respect to **20**. See Appendix 2 for ^{1}H NMR spectrum.

Reactions of IPrCuMn(CO)$_5$ (Figure 4a).

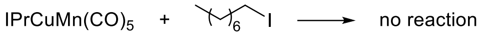

To a solution of IPrCuMn(CO)$_5$ (32.3 mg, 0.05 mmol) in THF (2 mL) was added 1-iodooctane (9 µL, 0.05 mmol). The reaction was stirred at 60 °C for 16 hours). The solvent was removed under reduced pressure, taken up in C_6D_6 and the reaction mixture analysed by ^{1}H NMR.

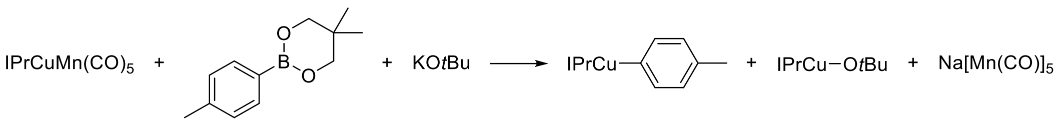

To a solution of IPrCuMn(CO)$_5$ (32.3 mg, 0.05 mmol) in THF (2 mL) was added NaO*t*Bu (48 mg, 0.5 mmol) and 4-tolylboronic acid neopentyl glycol ester (102 mg, 0.5 mmol). The reaction mixture was stirred at room temperature for one hour before the solvent was removed under reduced pressure. The residue was taken up in C_6D_6, filtered through a plug of Celite, and analysed by ^{1}H NMR. IPrCu(tol) and IPrCuO*t*Bu were found to be present in a ratio of 4.4:1. No IPrCuMn(CO)$_5$ was detected. See Appendix 3 for ^{1}H NMR spectrum.

Reaction of MeC(O)[Mn(CO)5] and IPrCu(tol) (Figure 4c).

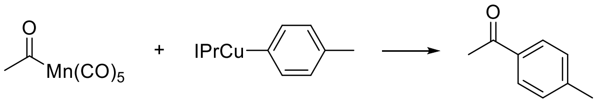

21

To a 22-mL scintillation vial was added [MeC(O)Mn(CO)$_5$] (23.8 mg, 0.1 mmol), IPrCu(tol) (54.3 mg, 0.1 mmol), and THF (2.5 mL). The vial was capped with a septum containing lid, and the septa pierced with a needle. The vial was placed within a solid, fitted, aluminium rack within a Parr pressure reactor and pressurised according to General Procedure A. The reaction was then heated to 60 °C for 16 hours. Upon completion of the reaction, decane (19 µL, 0.1 mmol) was added and an aliquot of the reaction mixture was taken and filtered through a silica plug, eluting with diethyl ether. GC analysis determined 4-methylacetophenone, **25**, to be present in 67% yield.

References

1. Banerjee, S.; Karunananda, M.K.; Bagherzadeh, S.; Jayarathne, U.; Parmelee S.R.; Waldhart, G.W.; Mankad, N.M. *Inorg. Chem.,* **2014***, 53,* 11307.

2. Motz, P.L.; Sheeran. D.J.; Orchin, M. *J. Organomet. Chem.*, **1990**, *383*, 201.

3. Santoro, O.; Collado, A.; Slawin, A.M.Z.; Nolan, S.P.; Cazin, C.S.J. *Chem. Commun*., **2013**, *49*, 10483.

4. Fujihara, T.; Xu, T.; Semba, K.; Terao, J.; Tsuji, Y. *Angew. Chem. Int. Ed.,* **2011**, *50*, 523.

5. Bidal, Y.D.; Lesieur, M.M.; Nahara, F.; Cordes, D.B.; Arachchine, K.S.A.; Slawin, A.M.Z.; Bertrand, G.; Cazin, C.S.J. *Adv. Synth. Catal.*, **2015**, *357*, 3155.

6. Berini, C.; Navarro, O. *Chem. Commun.*, **2014**, *48*, 1538.

7. Yoshida, Y.; Sakakura, Y.; Aso, N.; Okada, S.; Tanabe, Y. *Tetrahedron*, **1999**, *55*, 2183.

8. Collins, K.D.; Glorius, F. *Nat. Chem.,* **2013**, *5*, 597.

10. Sumino, S.; Ui, T.; Ryu, I. *Org. Lett.*, **2013**, *12*, 3142.

11. Wang, X.; Liu, M.; Xu, L.; Wang, Q.; Chen, J.; Ding, J.; Wu, H. *J. Org. Chem.* **2013**, *78*, 5273.

12. Hooper, J. F.; Young, R. D.; Weller, A. S.; Willis, M. C. *Chem. Eur. J.* **2013**, *19*, 3125.

Appendix 1: Reaction of IPrCuMn(CO)$_5$ with NaO*t*Bu and tolBneop

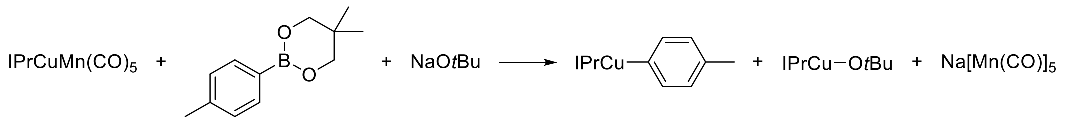

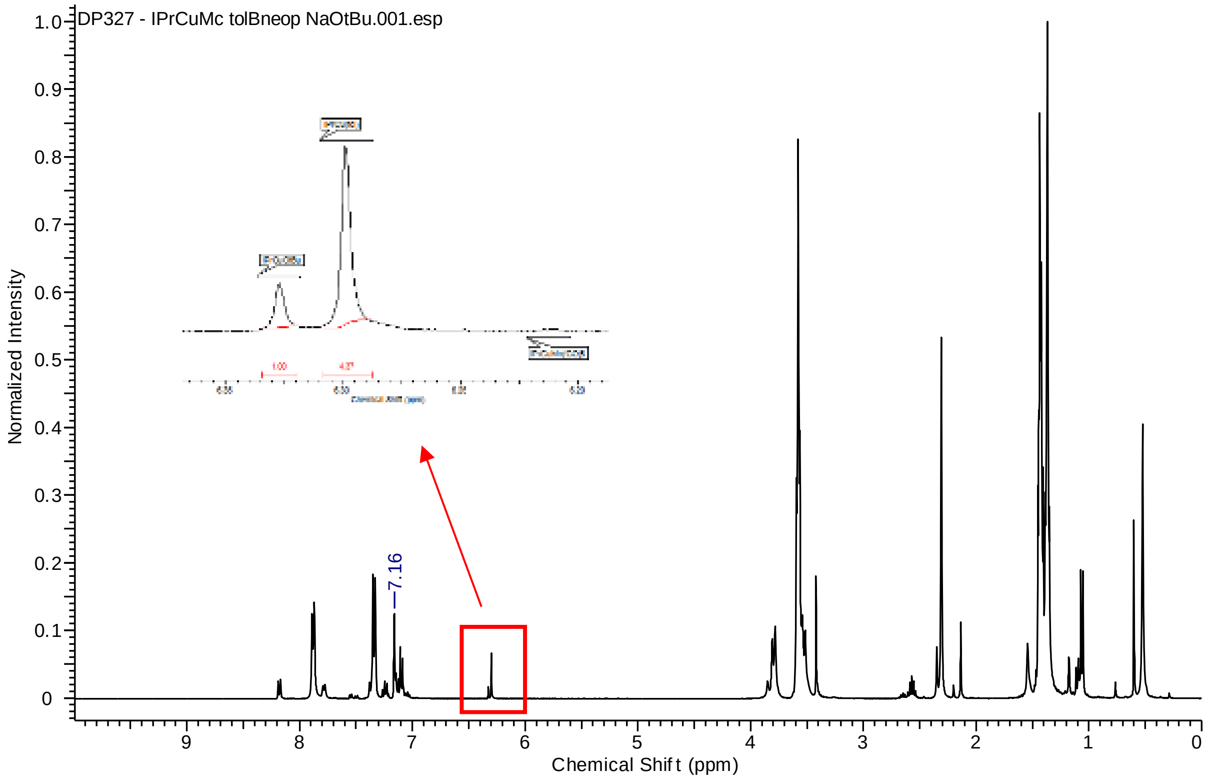

Appendix 2: NMR spectra of isolated compounds

1-(4-methoxyphenyl)-1-nonanone, 4

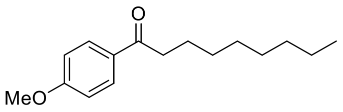

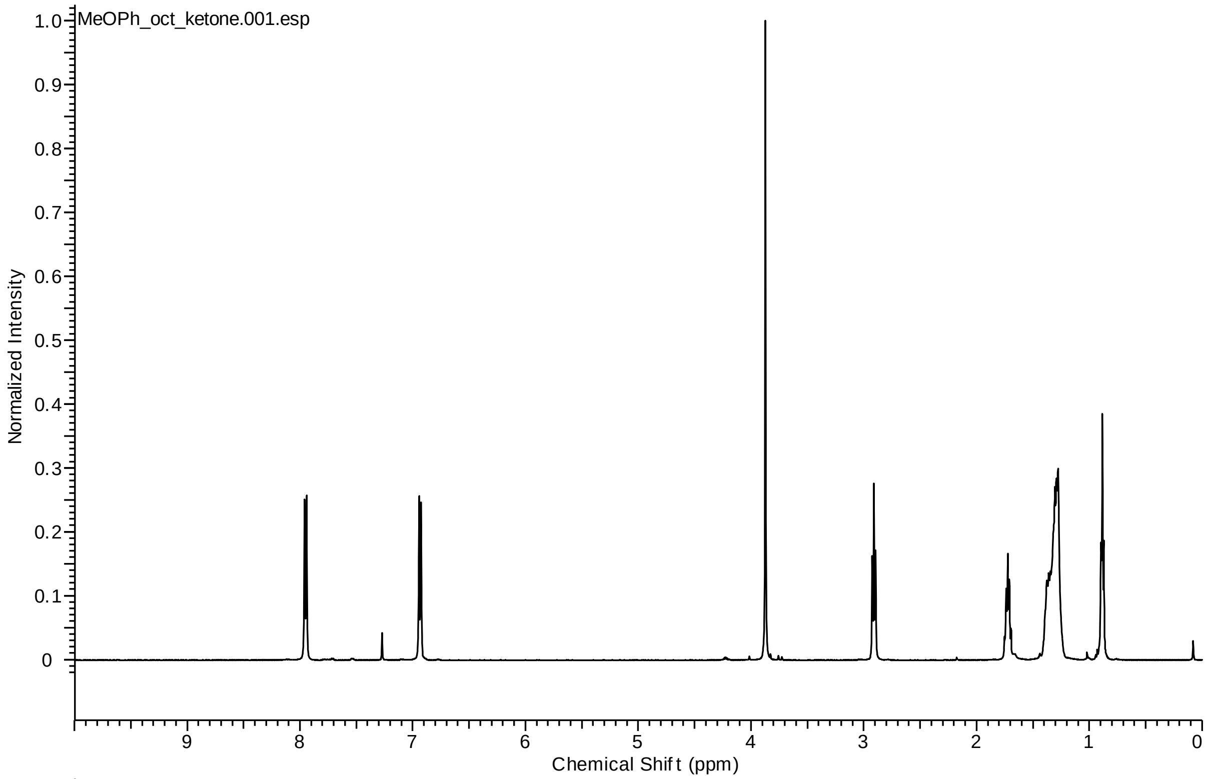

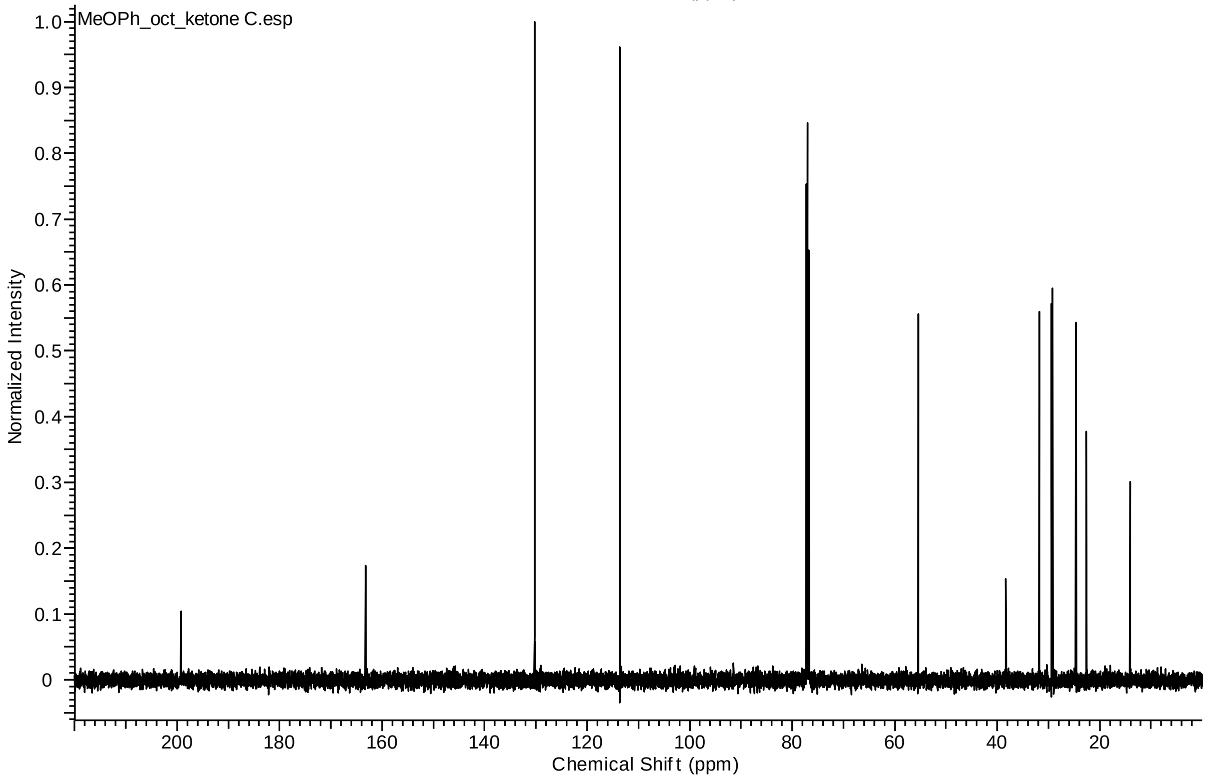

Methyl-(4-nonanoyl)benzoate, 7

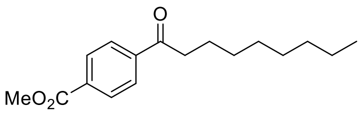

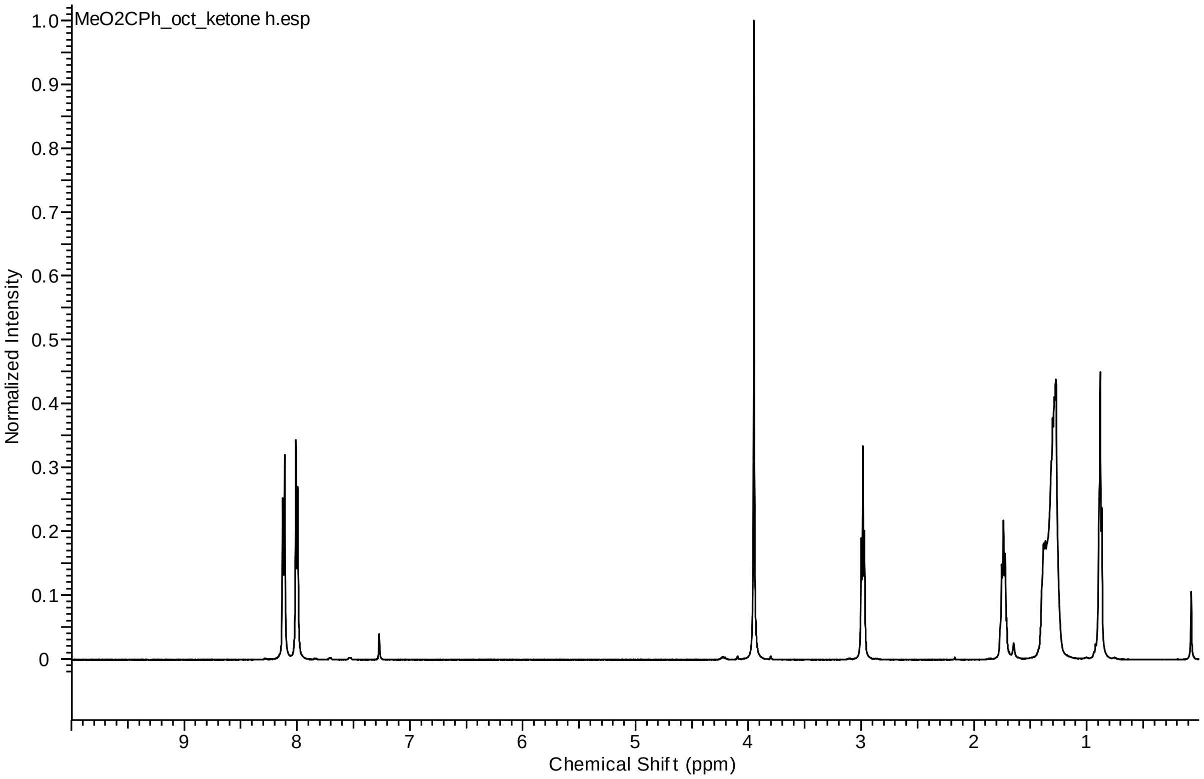

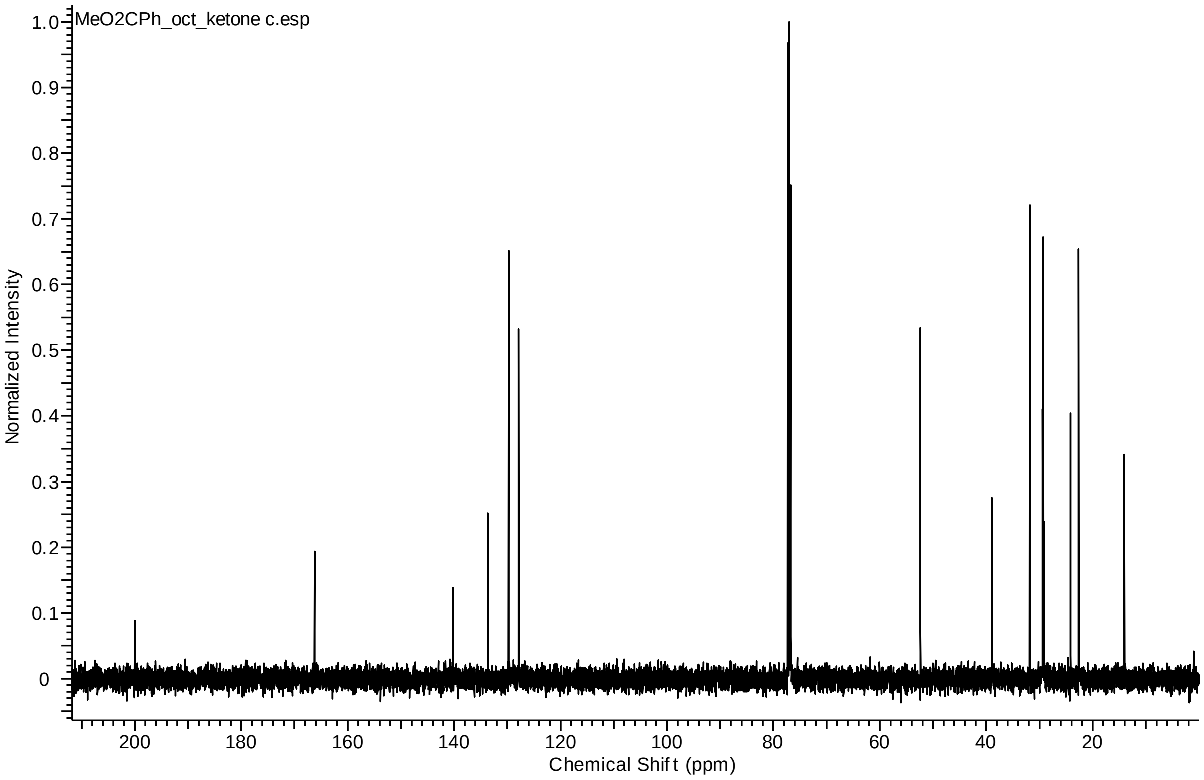

Methyl-(3-nonanoyl)benzoate, 6

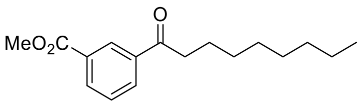

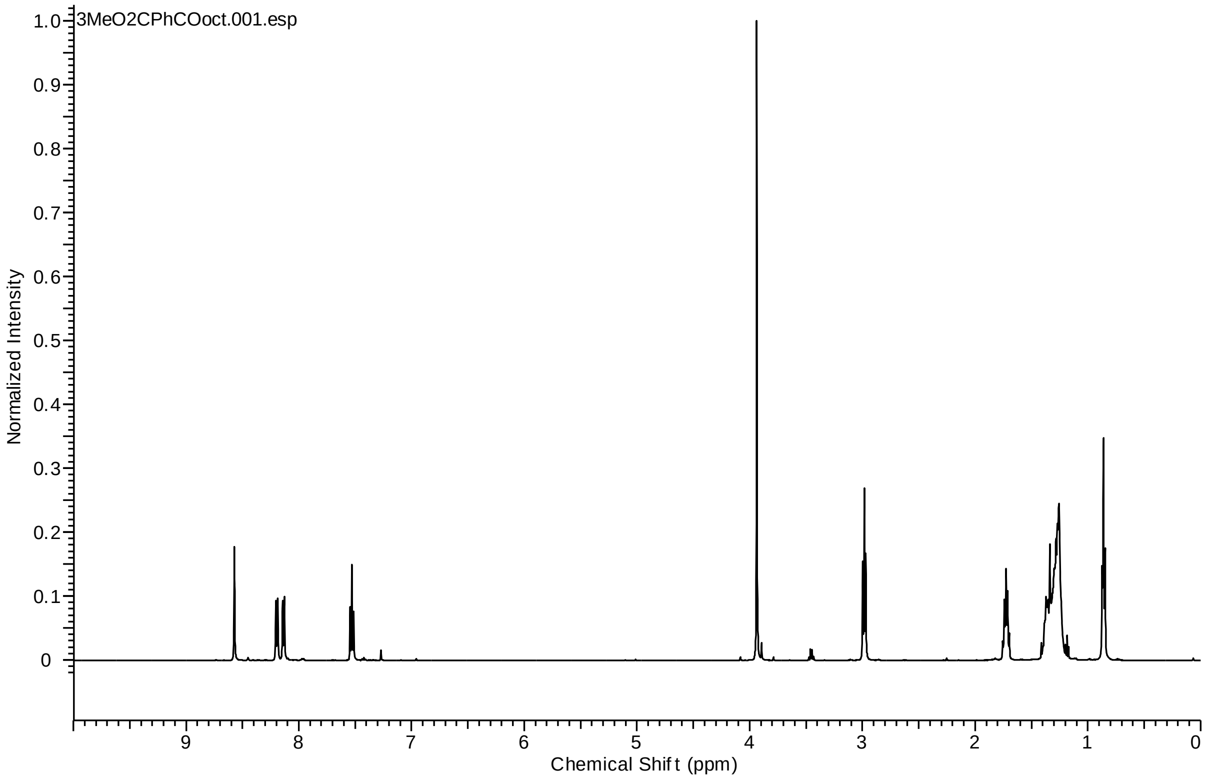

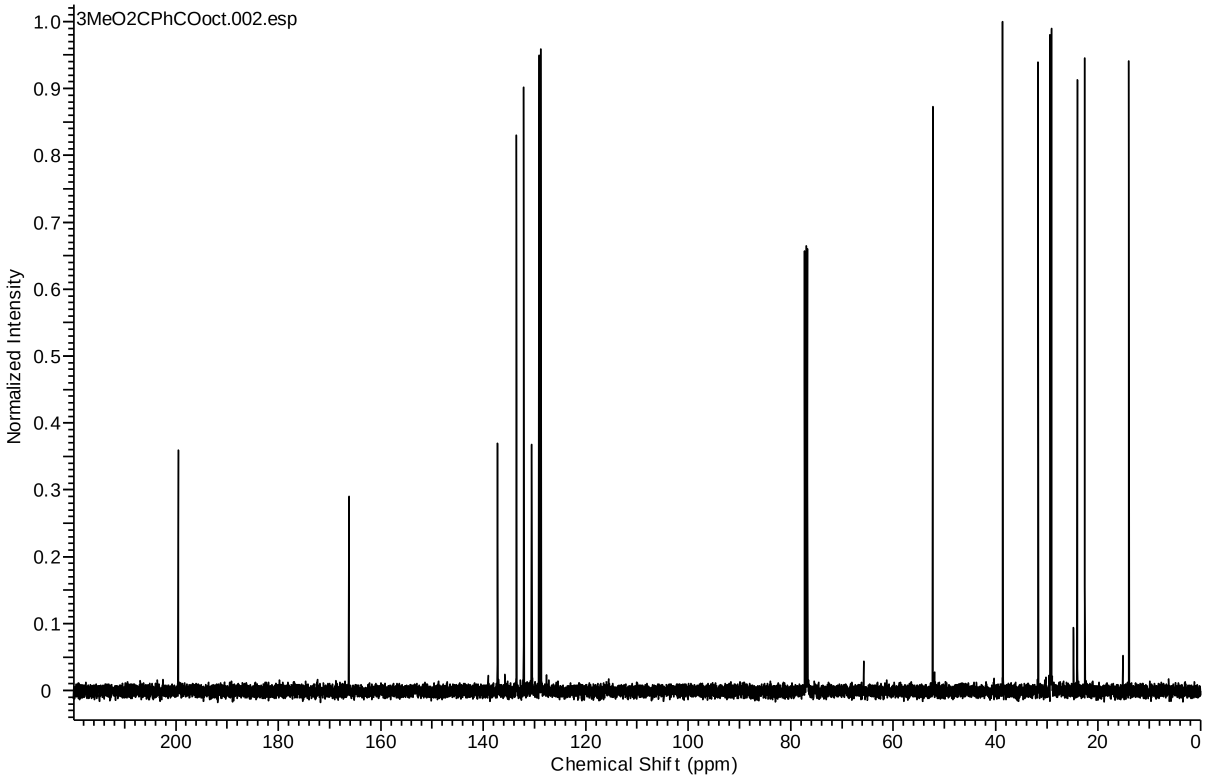

2-Methyl-1-(4-methylphenyl)-1-propanone, 15.

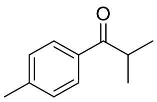

10

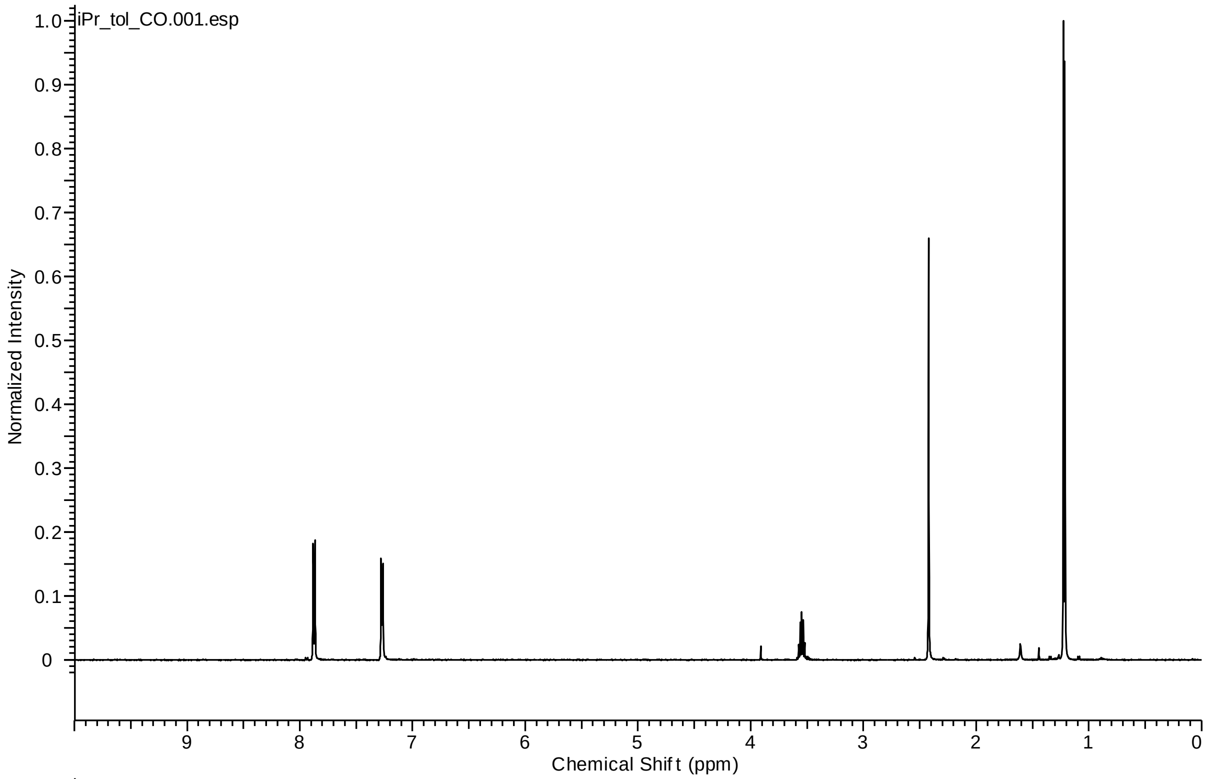

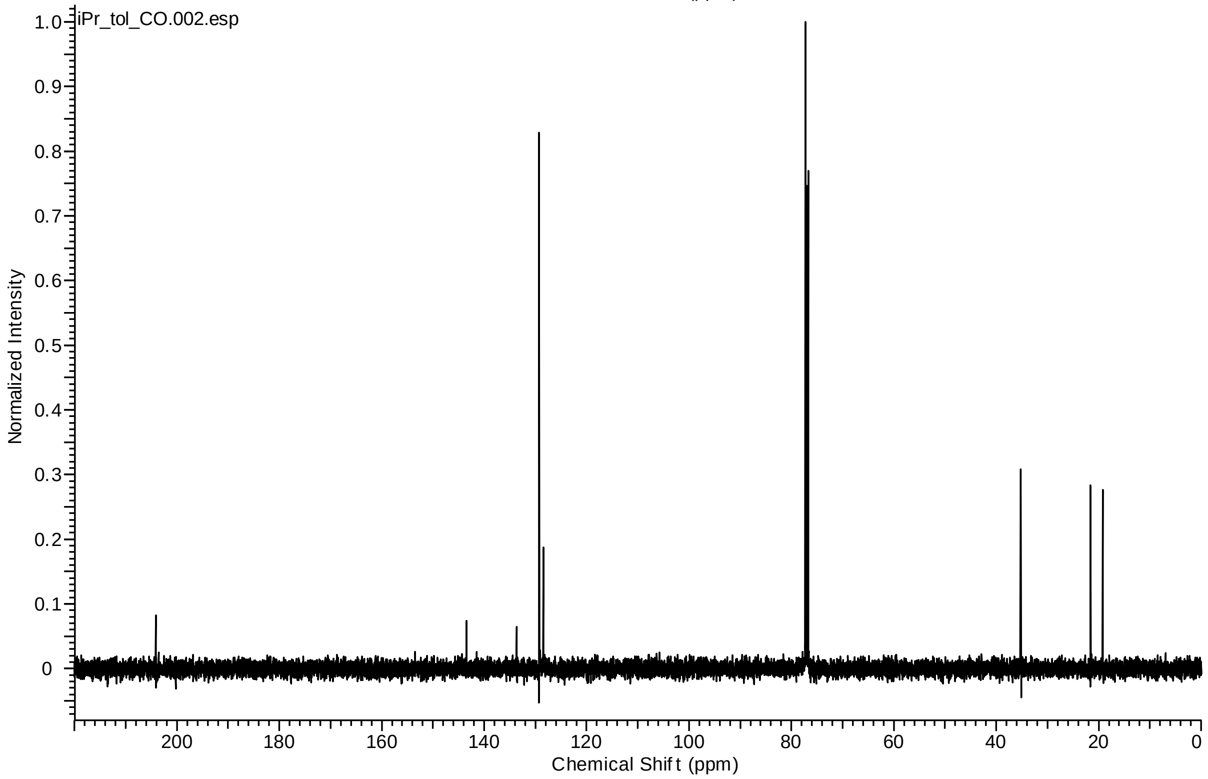

1-Cyclohexyliodoethane.

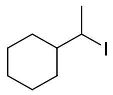

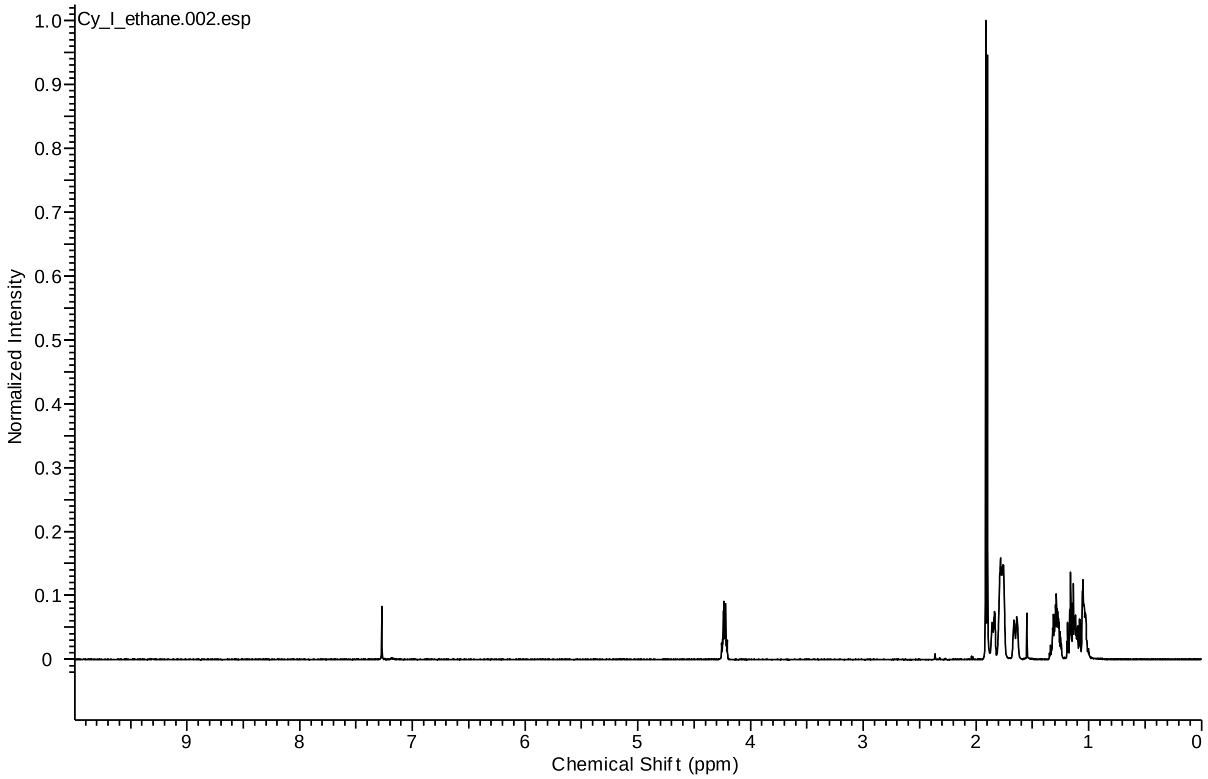

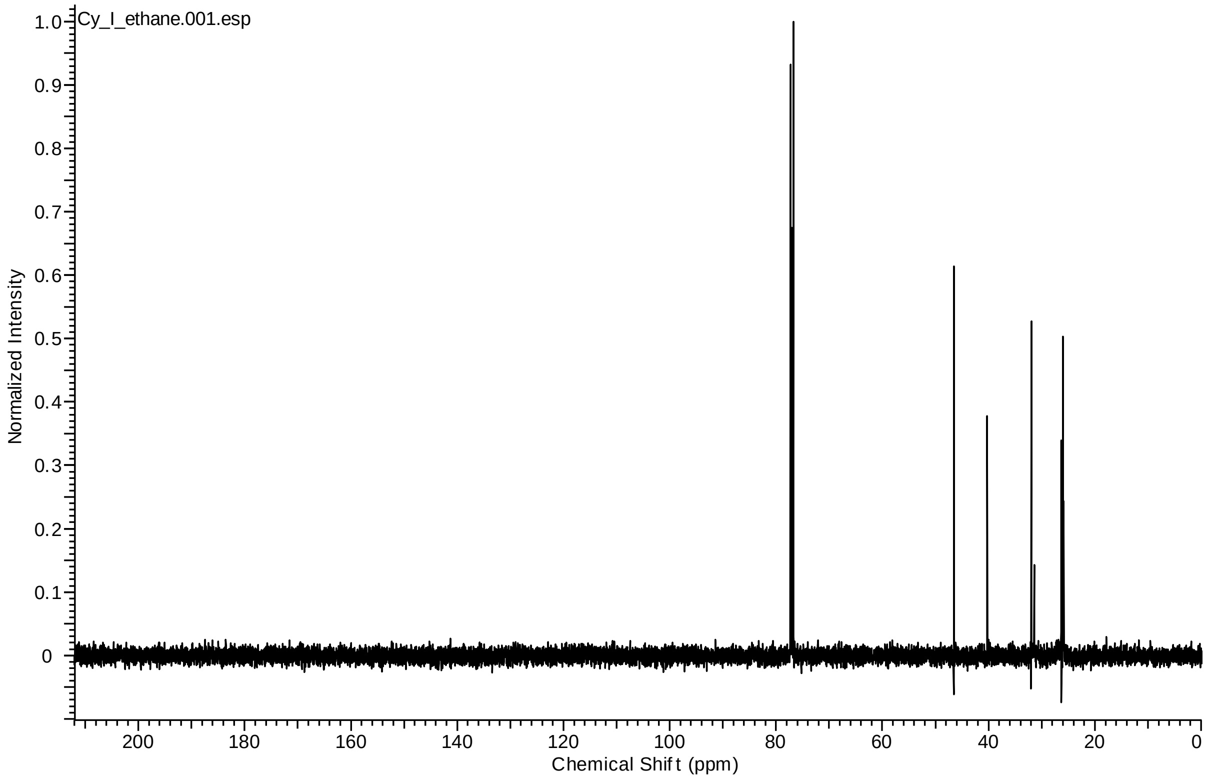

2-Cyclohexyl-1-(4-methylphenyl)-1-propanone, 16.

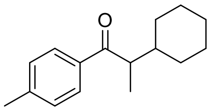

11

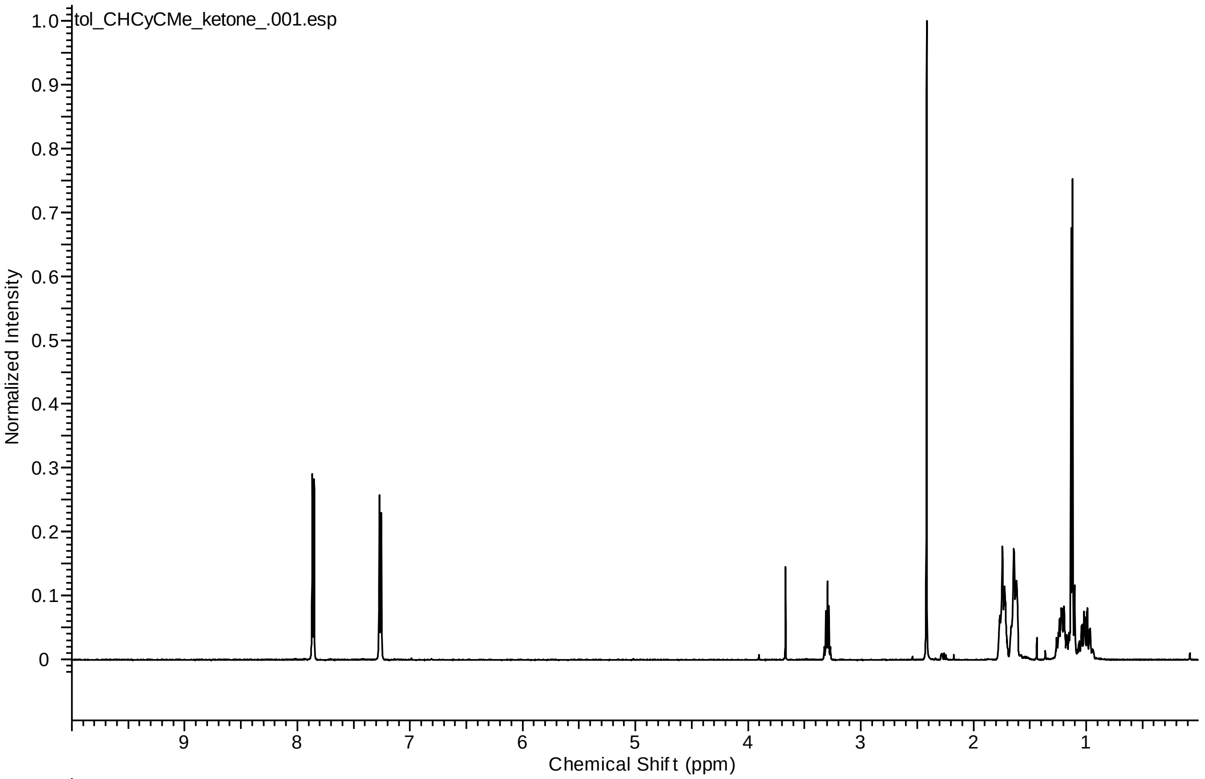

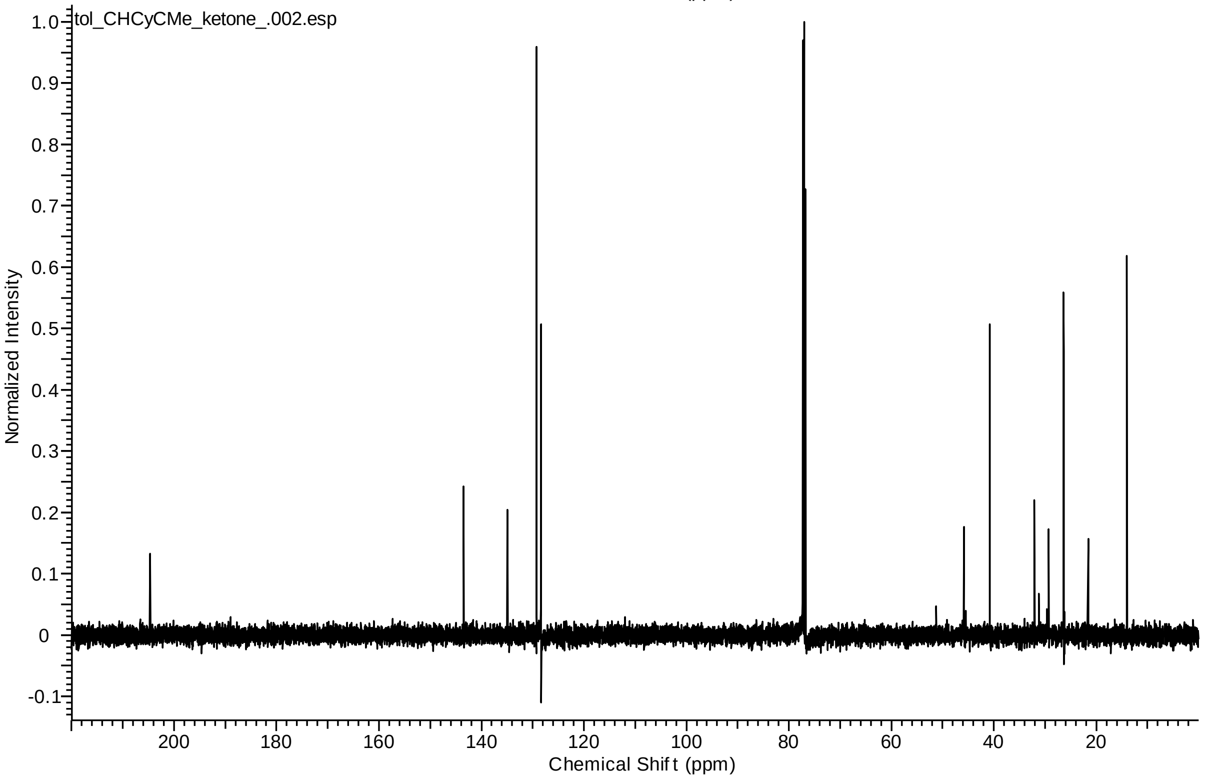

(3,5-Dimethoxyphenyl)-1-nonanone, 5

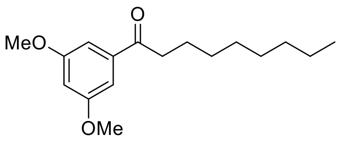

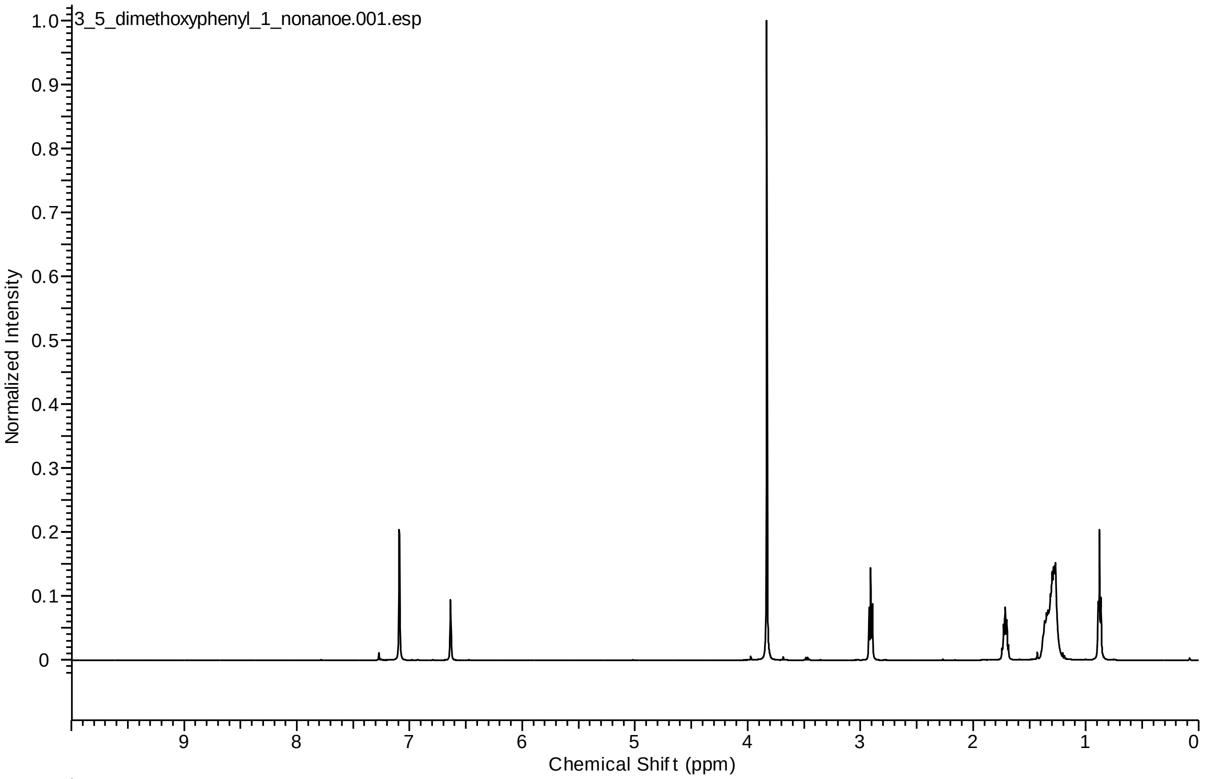

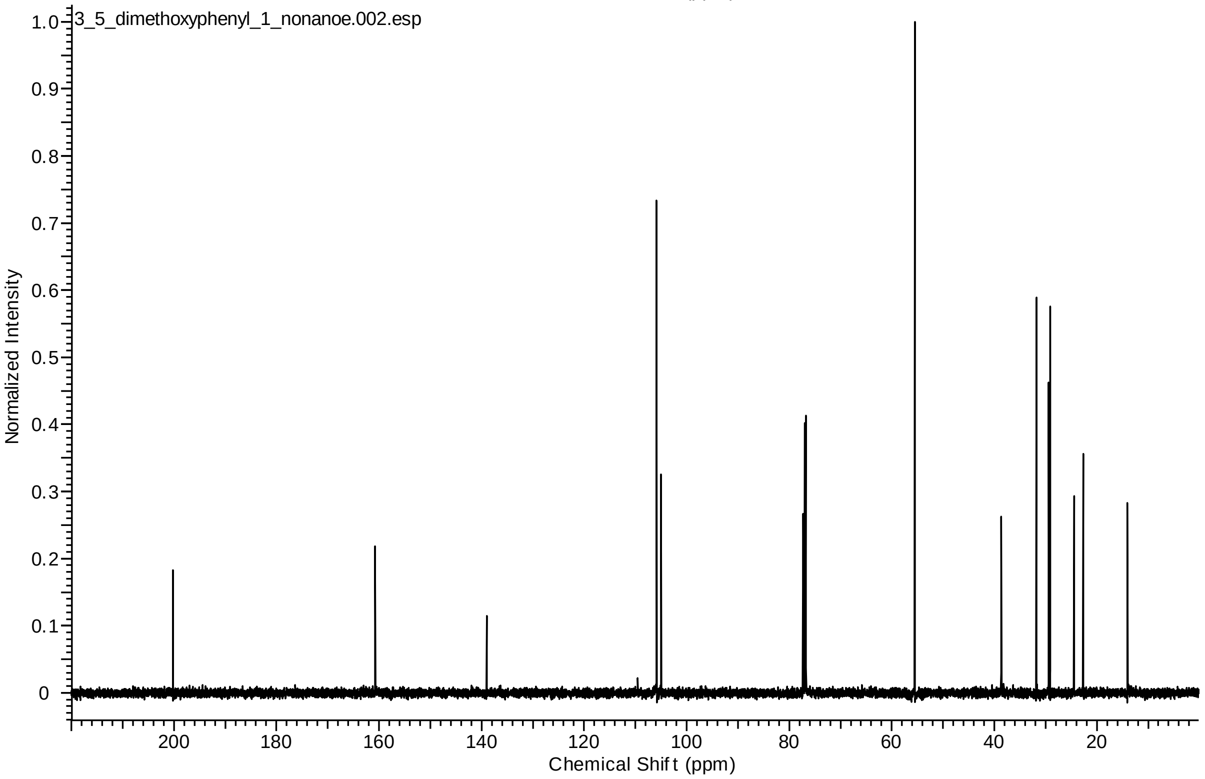

(4-Trifluoromethylphenyl)-1-nonanone, 9

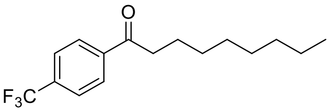

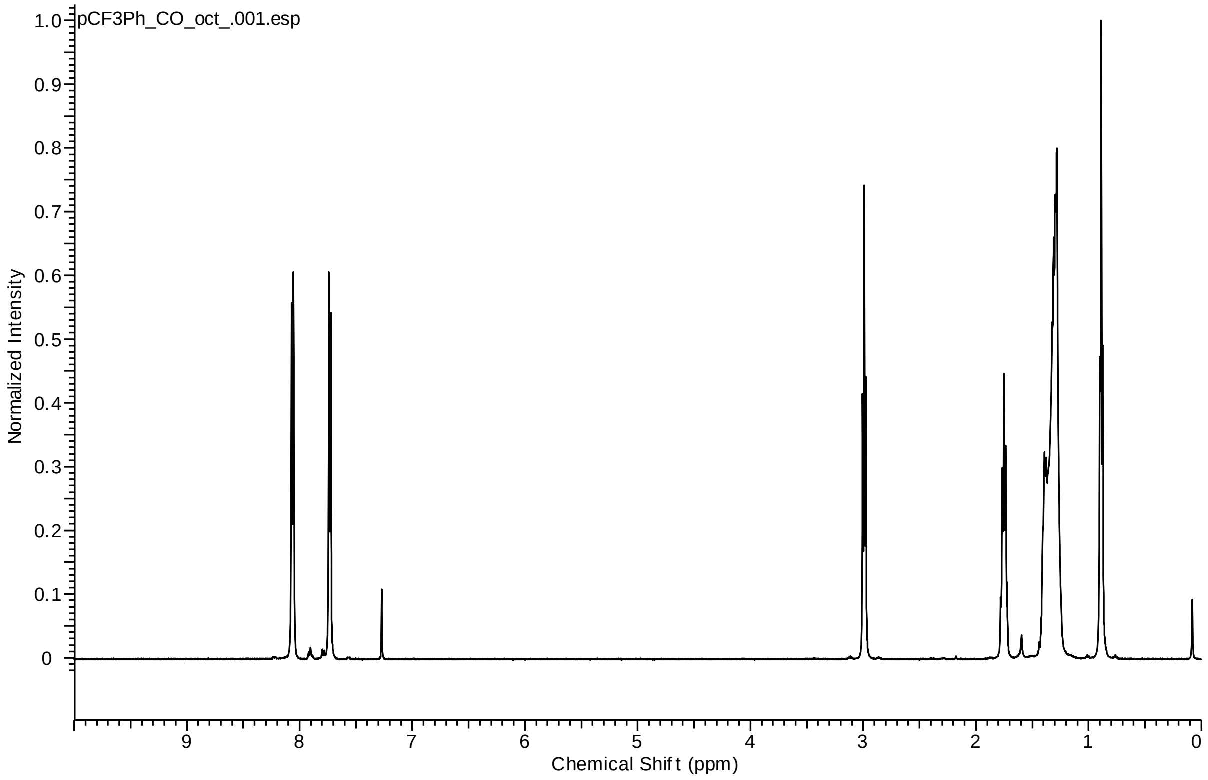

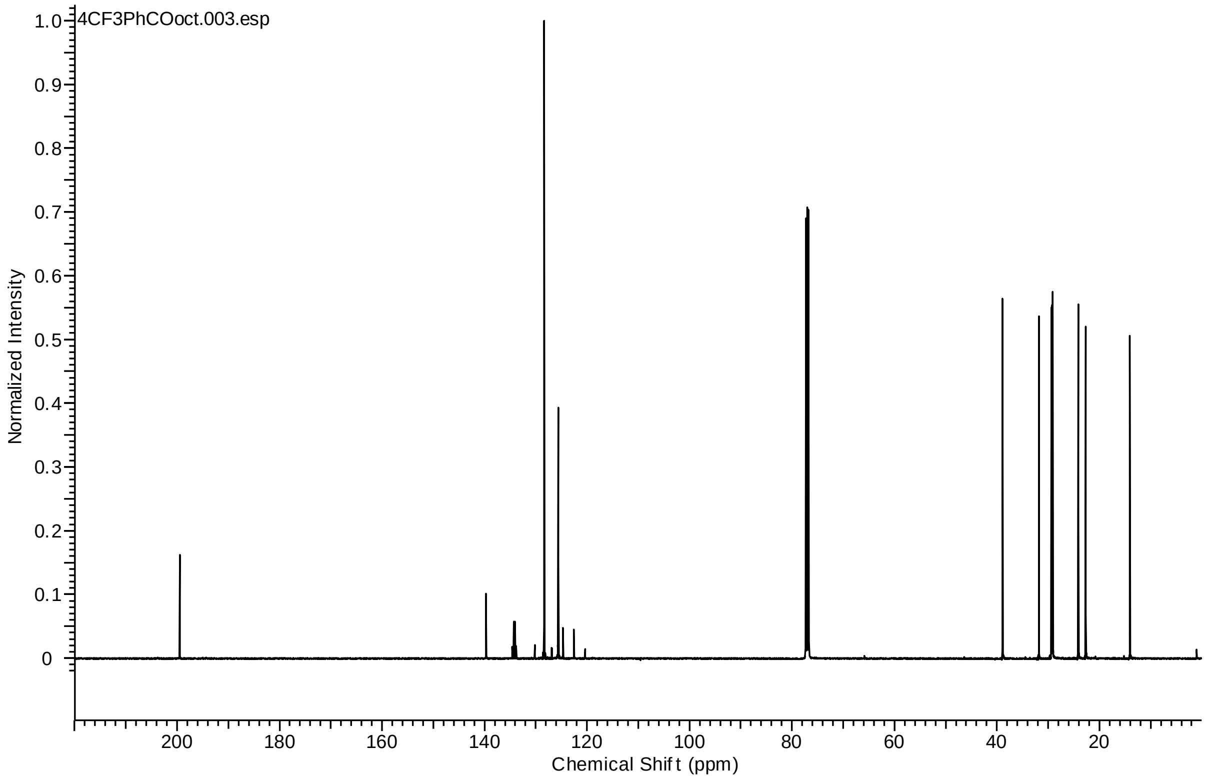

(2-Fluorophenyl)-1-nonanone, 11

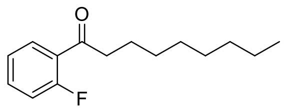

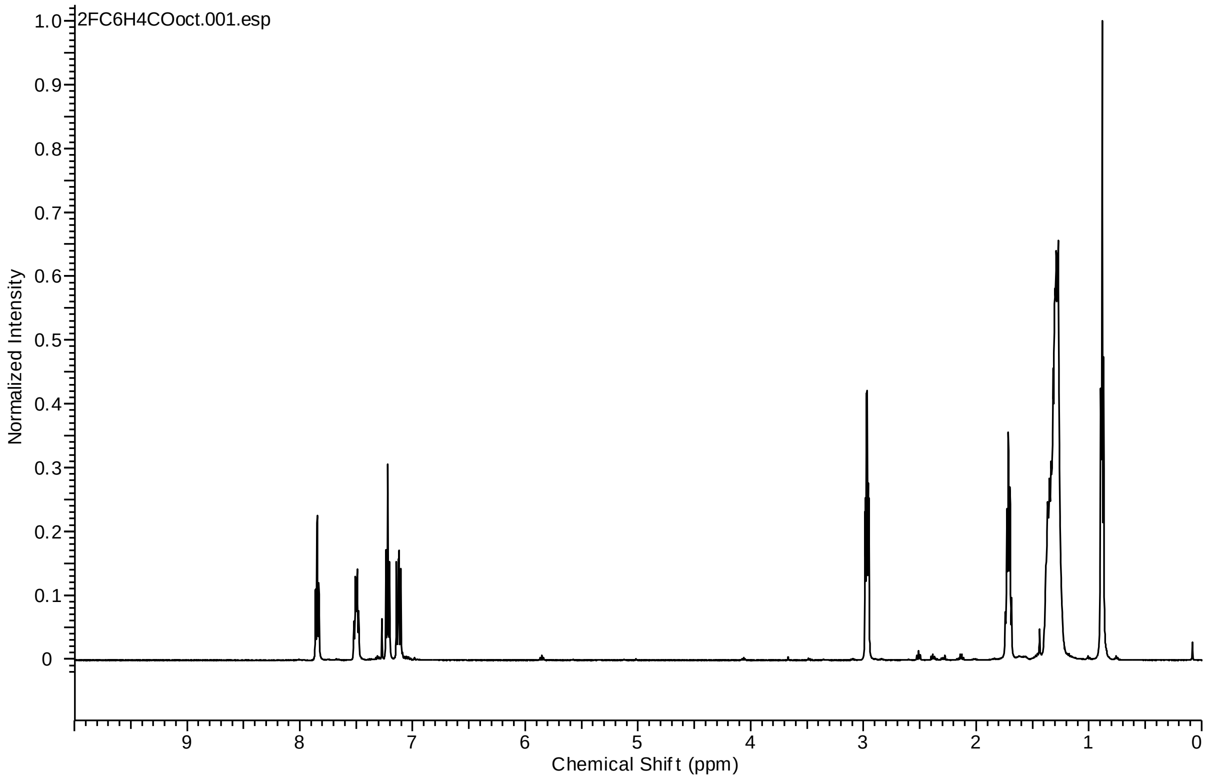

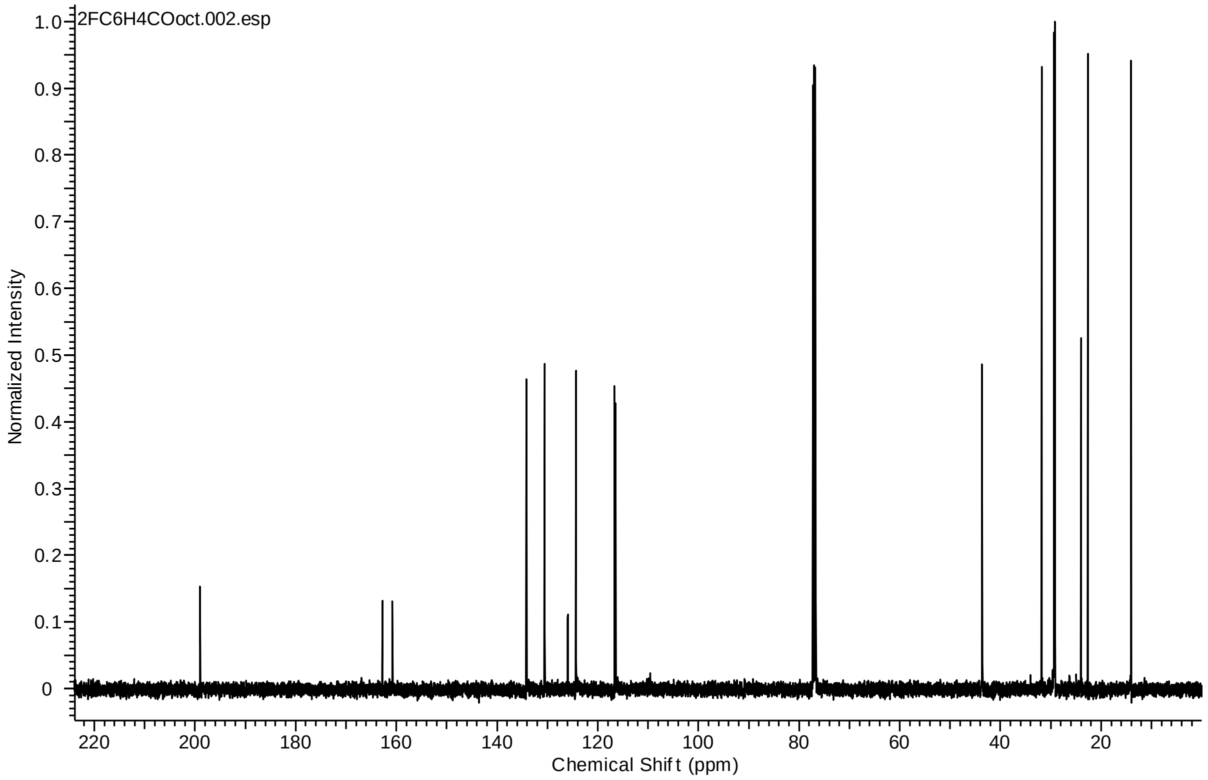

4-Nonanoylbenzonitrile, 10

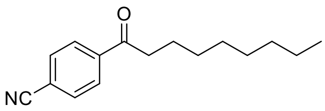

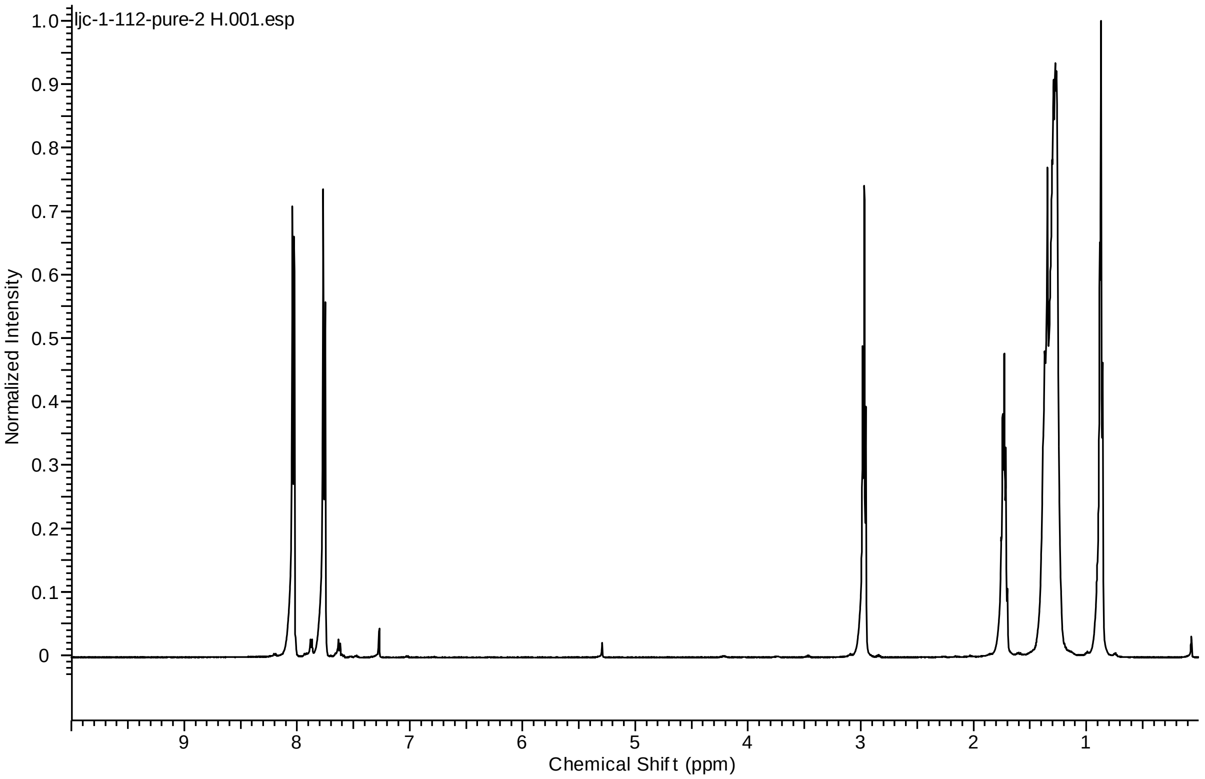

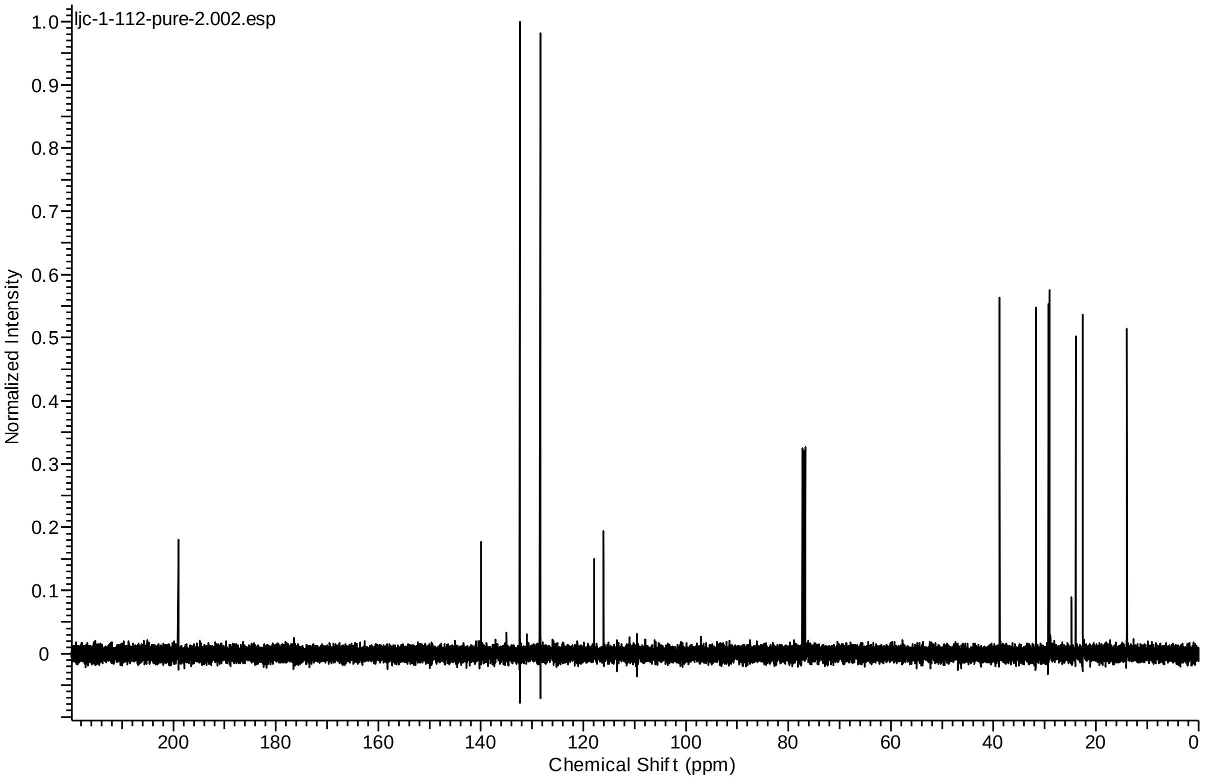

(3-Trimethylsilyl)-1-nonanone, 19

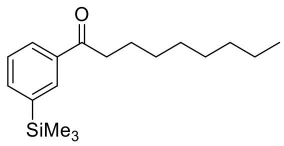

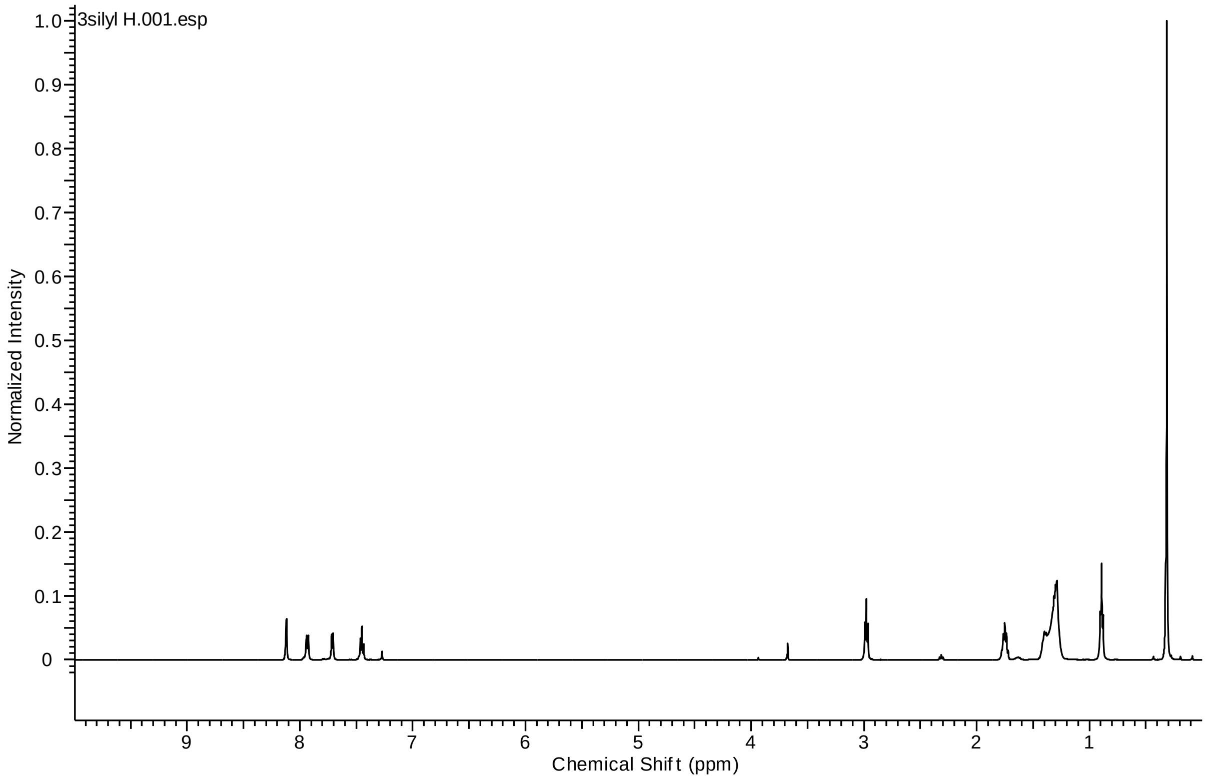

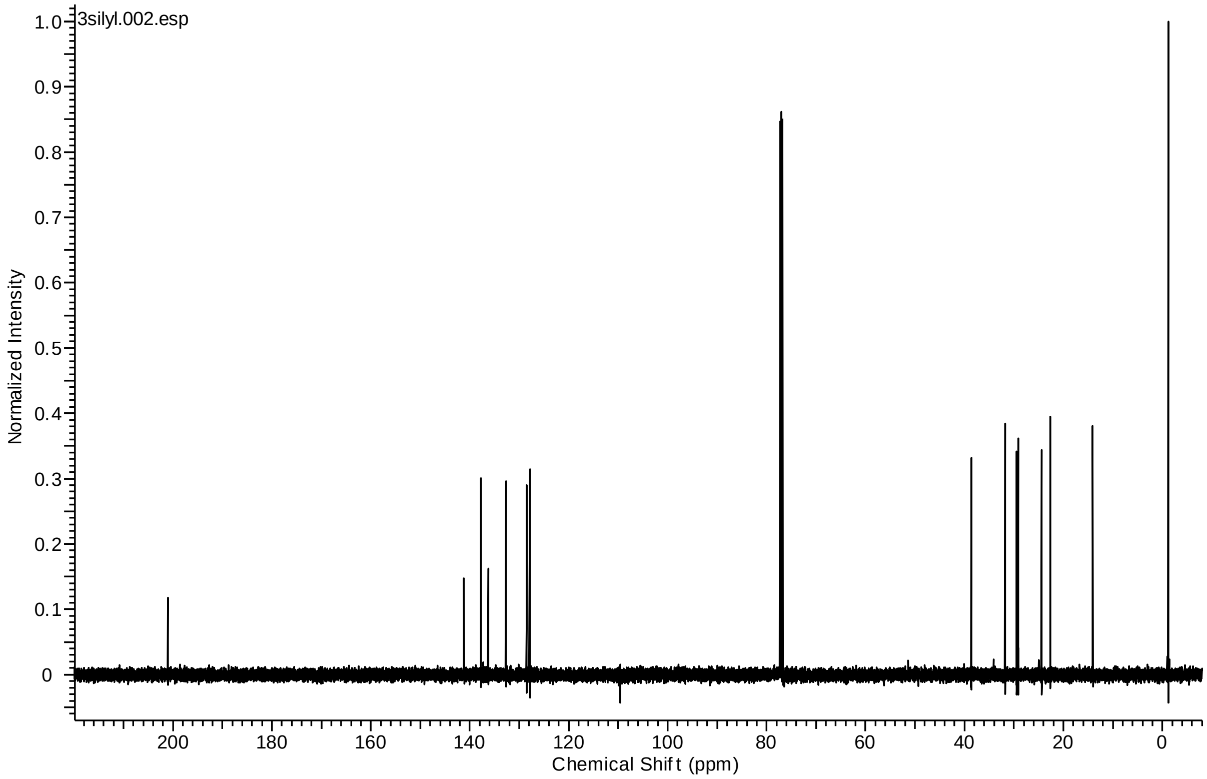

N,N-Dimethyl-4-nonanoylbenzamide, 8

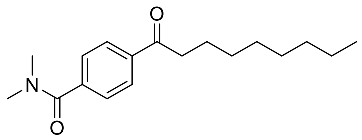

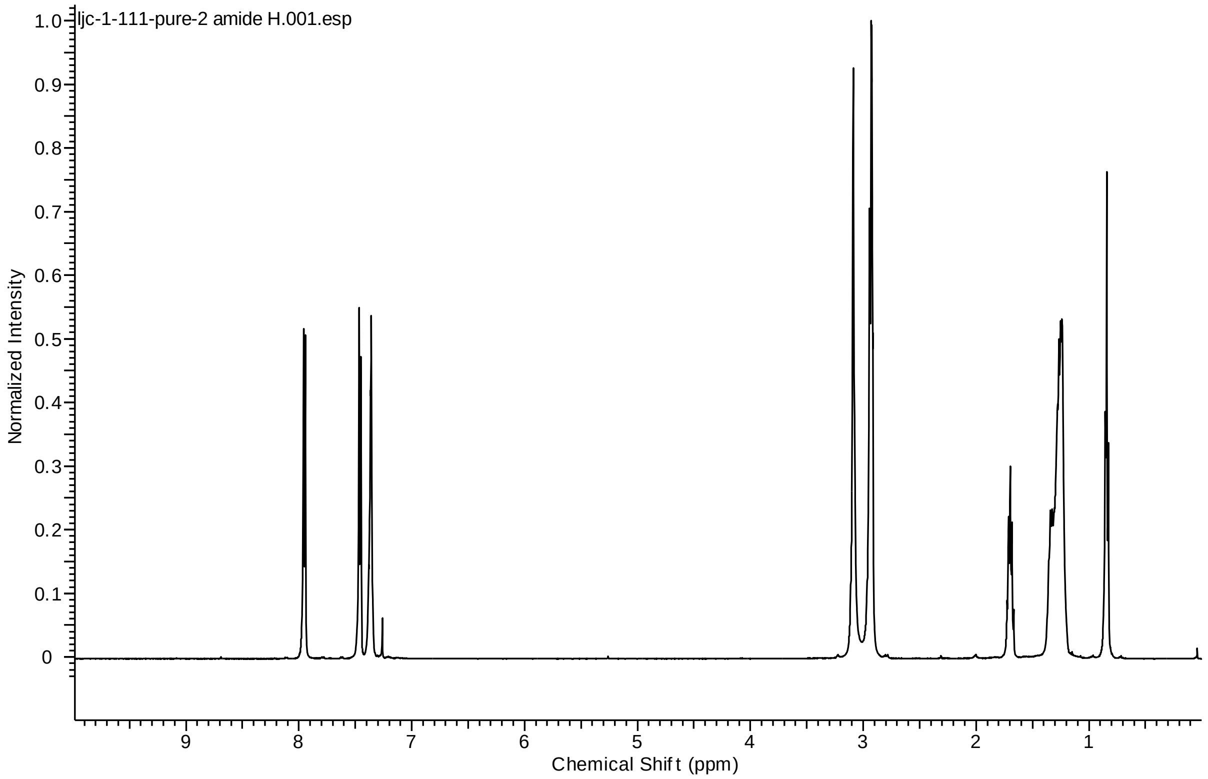

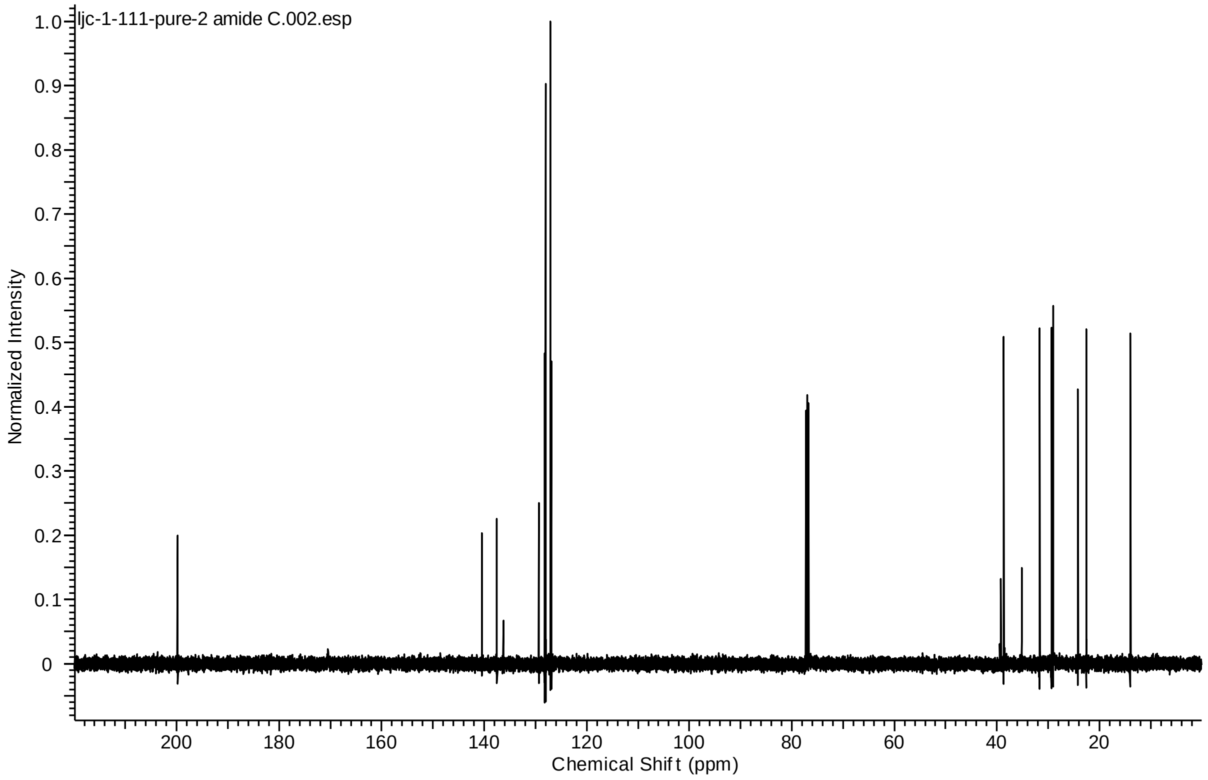

1-(4-bromophenyl)-1-nonanone, 17.

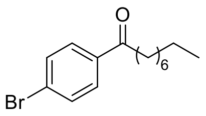

6

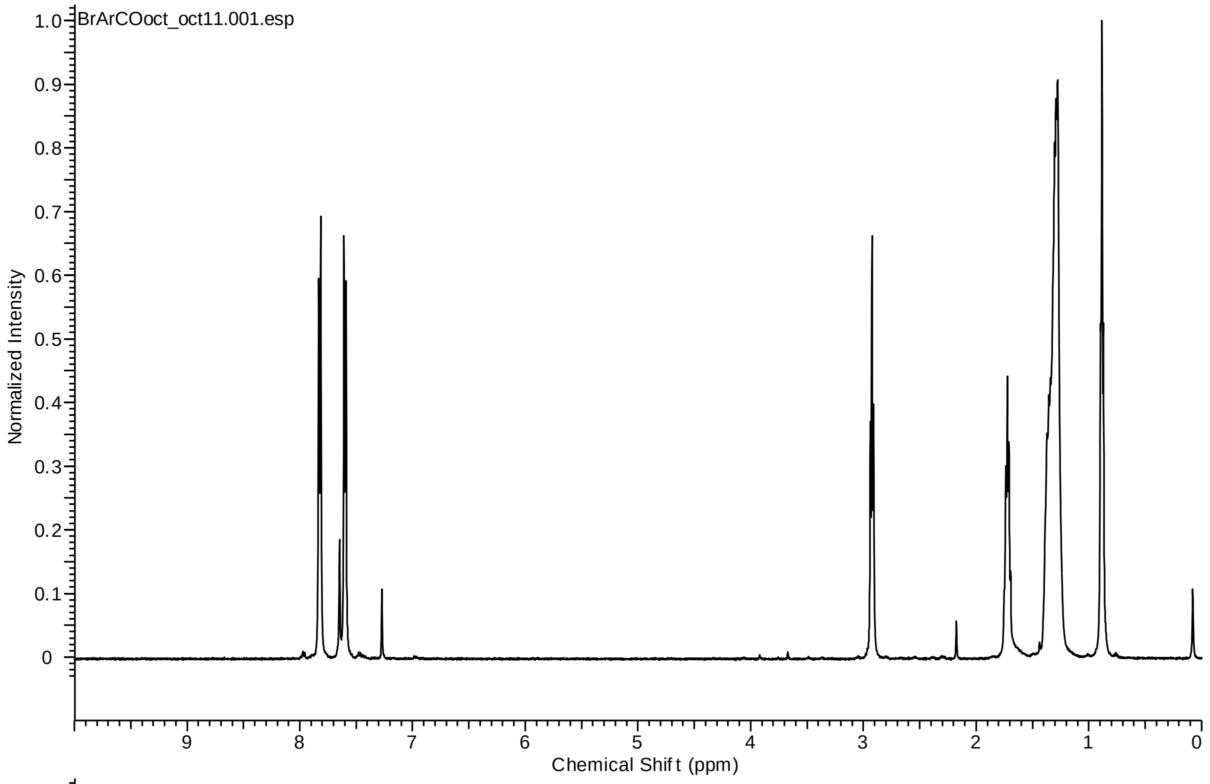

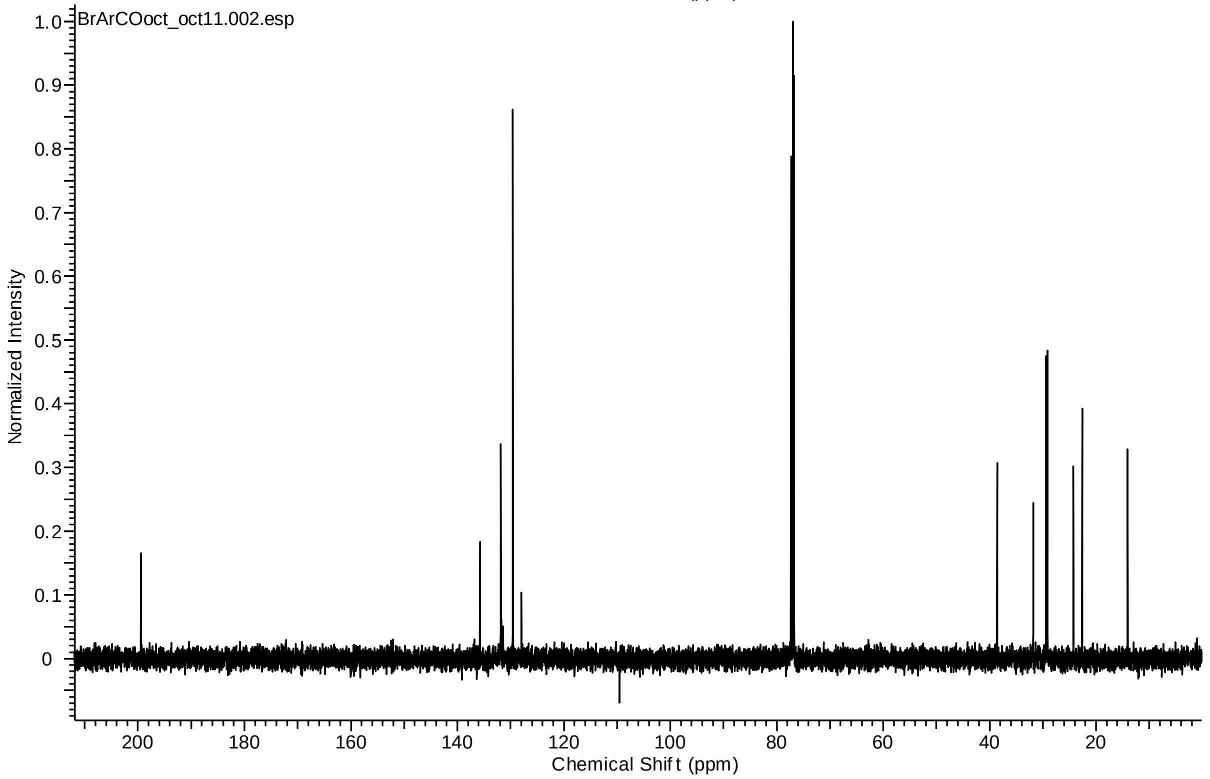

Appendix 2: Pd catalyzed coupling of 17

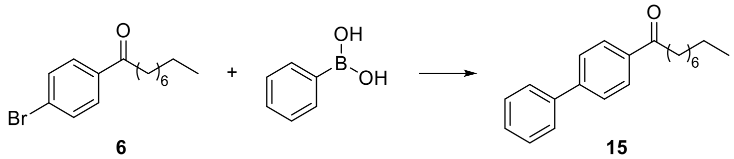

6 15

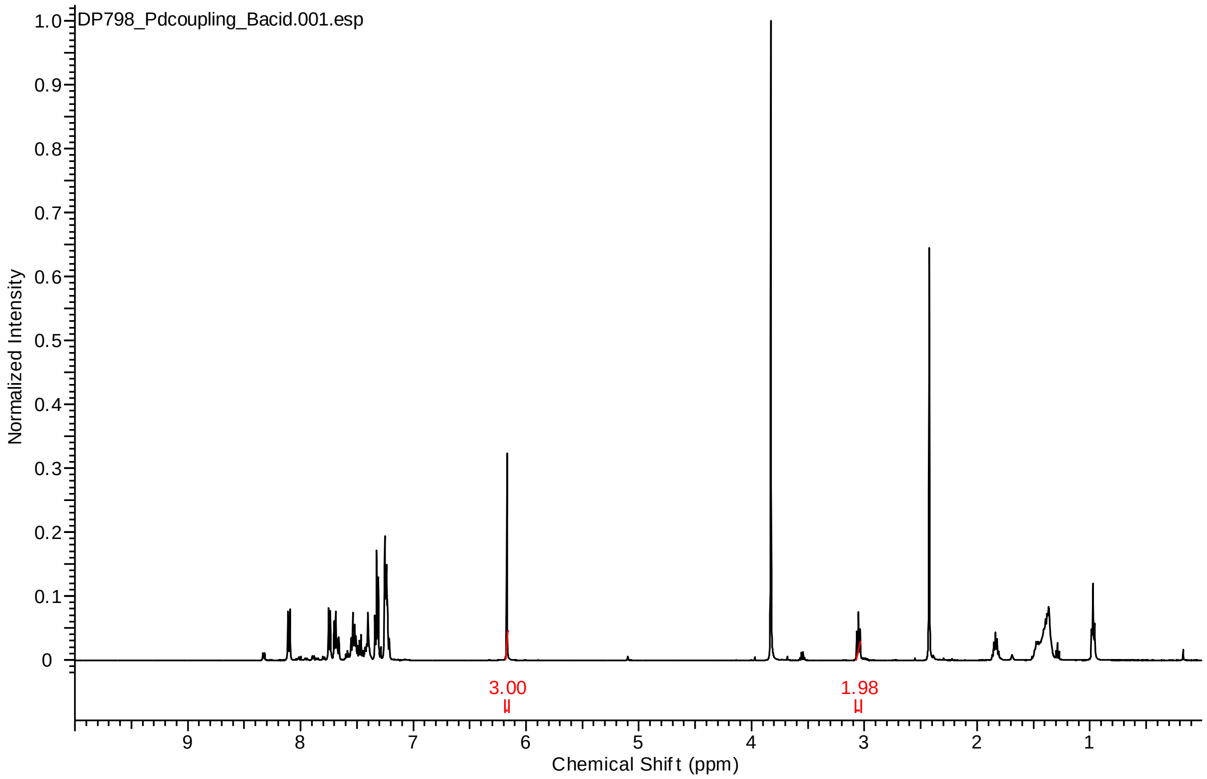

Appendix 3: Reductive amination of 21

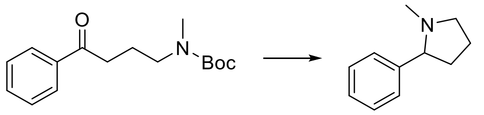

17 **18**

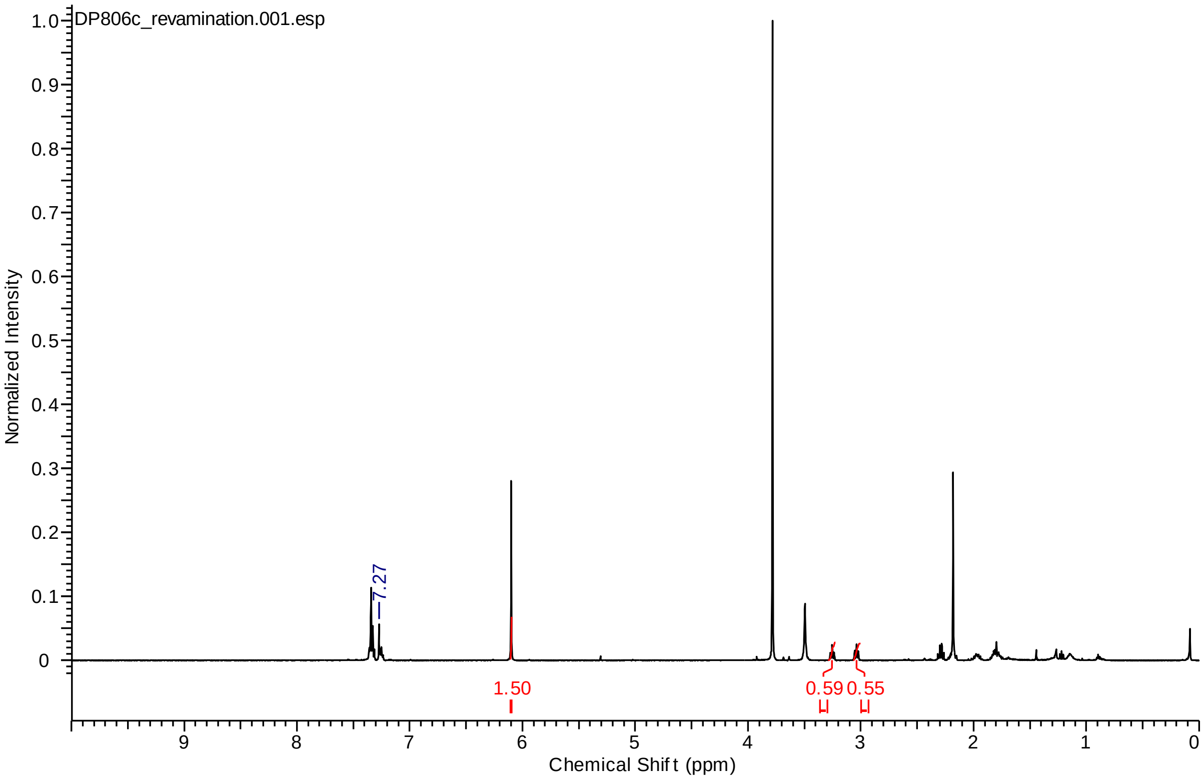

Supplement: Supplementary file 1 [file SC-008-C7SC01170A-s001.pdf]
